# Recent advances in nanocellulose scaffold membranes: Sources, processing and functionalization

**DOI:** 10.1016/j.mtbio.2026.103221

**Published:** 2026-05-07

**Authors:** Hanyuan Chen, Yanqun Huang, Feng Wang, Ziyan Fu, Ao Li, Malaz Abdelsadig, Mark Brassil, Yan Xia, Bei Zhou, Guanben Du, Mizi Fan

**Affiliations:** a Nanocellulose and Biocomposites Research Centre, College of Engineering, Design and Physical Sciences, Brunel University London, United Kingdom; b College of Material Engineering, Fujian Agricultural and Forestry University, China; c Yunnan Provincial Key Laboratory of Wood Adhesives and Glued Products, International Joint Research Centre for Biomaterials, Southwest Forestry University, China; d Smart Reactors, Galway Technology Park, Parkmore Road, Galway, Ireland

**Keywords:** Nanocellulose membranes, Sources, Processing technologies, Surface modification, Biomedical applications

## Abstract

Nanocellulose, derived from renewable cellulose resources, has emerged as a highly promising candidate for biomedical scaffold membrane applications owing to its excellent mechanical properties, tunable surface chemistry, biodegradability, and biocompatibility. The performance of nanocellulose-based membrane materials can be significantly enhanced through the integrated regulation of raw material sources, processing and functionalization. This review provides a comprehensive overview of the advances in nanocellulose scaffold membranes from raw material sources, processing technologies, functionalization strategies and biomedical applications. The review especially focuses on how to synergistically integrate these parameters to achieve a balanced design for customizable membranes. Furthermore, a design-oriented conceptual framework for the fabrication of regenerated nanocellulose composite membranes by electrospinning is discussed, which can provide guidance for future material and process development. Despite the preliminary progress achieved to date, several critical bottlenecks continue to hinder practical implementation, including difficulties in pore-structure regulation, long-term biosafety assessment, standardized large-scale manufacturing, and cost-effective production. Overall, this review not only summarizes the latest advancements in nanocellulose-based scaffold membranes, but also points out a future direction for their rational design and biomedical translation.

## Highlights

• A balanced design strategy for nanocellulose scaffold membranes from source, processing and functionalization is proposed.
• The central performance conflicts that constrain biomedical scaffold membrane design are emphasized.
• The regenerated nanocellulose composite membranes as a tunable design framework for future biomedical applications is positioned.

## Graphical abstract

**Figure:**
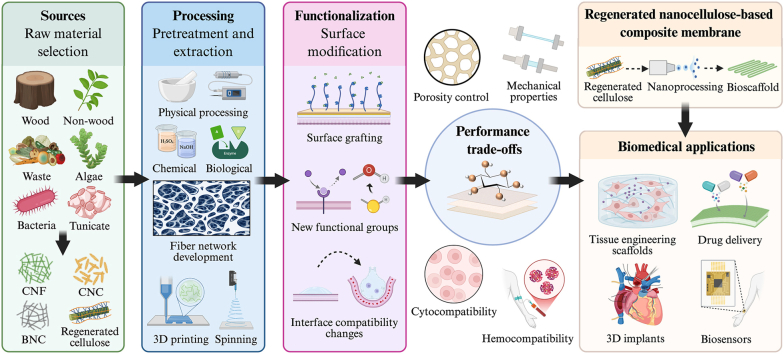

## 1 Introduction

Advancements in nanotechnology have significantly accelerated the development of advanced biomaterials for biomedical applications. Nanocellulose, a nanoscale material derived from natural cellulose, has at least one dimension below 100 nm. It stands out among various nanomaterials due to its unique physical, chemical, and biological properties. These include high crystallinity, large surface area, mechanical robustness, and barrier performance, as well as favorable attributes such as chemical reactivity, biocompatibility, biodegradability, and low cytotoxicity [[1], [2], [3], [4]]. Owing to these advantages, nanocellulose and its composites have been widely explored in diverse advanced biomedical applications [5,6], such as tissue engineering (e.g., bio-scaffolds and bone regeneration), drug delivery, wound dressings, vascular grafts, and biosensors, and serve as ideal materials for the manufacture of advanced medical devices [4,[7], [8], [9]]. In particular, the structural tunability of nanocellulose makes it a highly promising candidate material for scaffold-based biomedical systems [[10], [11], [12]].

Despite these advantages, transitioning raw nanocellulose into high-performance scaffold membranes remains a complex challenge. At present, it is difficult to control the pore structure and long-term biocompatibility of nanocellulose scaffold membranes in the biomedical field solely through surface functionalization or modified processing techniques. More importantly, current studies tend to treat the sources, processing techniques, and functionalization strategies of nanocellulose as independent variables without systematically integrating these factors. In practical applications, however, the performance of nanocellulose-based scaffold membranes is governed by the synergistic effects of raw material selection, processing techniques, and functionalization strategies. Additionally, challenges related to scalability, cost, and biosafety assessment continue to hinder clinical translation and large-scale production [13]. In the future, a more customized design approach for different types of nanocellulose, including additive manufacturing, nanomanufacturing, and biomanufacturing, should be explored to improve the comprehensive properties of nanocellulose-based products [14].

**Fig. 1:**
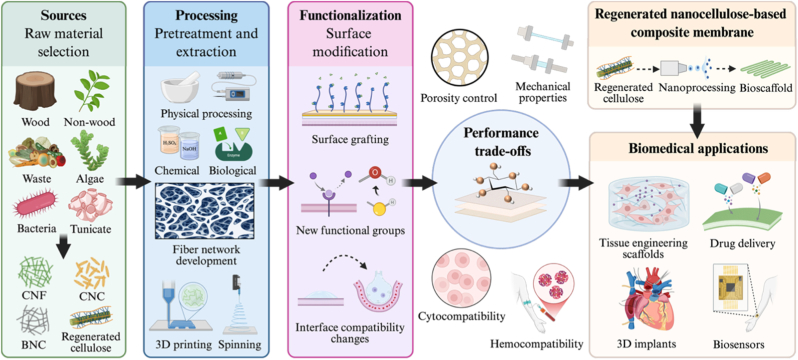
Integrated Design Framework of Nanocellulose-Based Scaffold Membranes for Biomedical Applications: From sources, processing to functional Integration.

This review provides a comprehensive overview of nanocellulose-based scaffold membranes from the perspectives of source, processing, and functionalization, focusing on their synergistic roles in achieving balanced design for biomedical applications and highlighting the material-process-functionalization relationship that determines membrane performance. Furthermore, the paper proposes a concept and design-driven strategy for the preparation of regenerated nanocellulose-based electrospun membranes, aiming to provide guidance for future material and process optimization. Fig. 1 illustrates the roadmap for customized design of nanocellulose-based scaffold membranes for biomedical applications. By integrating the characteristics of basic materials, preparation techniques, and modification strategies with application requirements, this paper seeks to provide a clearer design roadmap for nanocellulose-based scaffold membranes used in the biomedical field.

## 2 Sources and classification of nanocellulose

### 2.1 Sources of nanocellulose

**Fig. 2:**
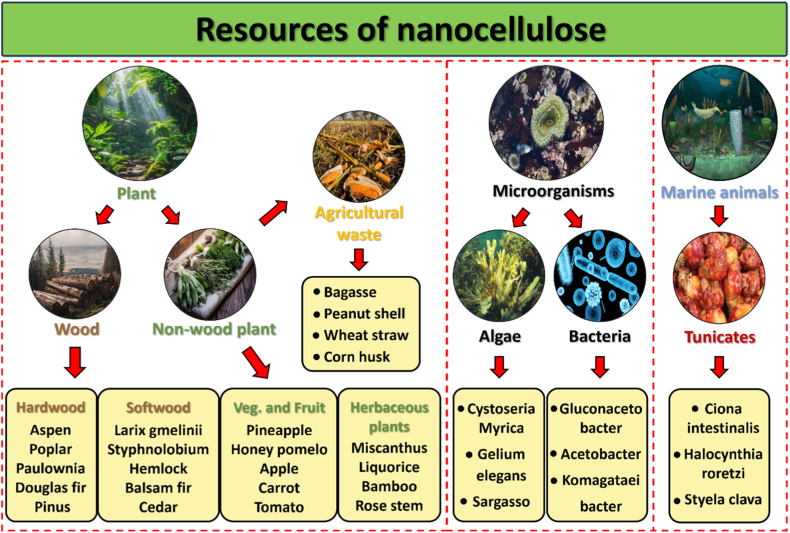
Summary of nanocellulose sources (Images sourced from iStock, Alamy, Prasanth Kv/Unsplash).

Natural fibers are the main source for extracting nanocellulose, which can generally be divided into plant-based fibers, animal-based fibers and mineral-based fibers [15]. Among these, plant fibers are preferred for biocomposite production because animal fibers are costly, and mineral fibers raise health concerns [16]. Recently, various sources for nanocellulose production, including plants, bacteria, algae, and animals, have been extensively studied [16,17]. Fig. 2 shows the various sources of nanocellulose. Plant-derived nanocellulose, the most common type, is typically categorized into wood-based and non-wood-based sources. Additionally, agricultural by-products and food wastes, such as rice husks, wheat straw and pineapple peel, are increasingly utilized as alternative, sustainable cellulose sources that also address waste management challenges [18,19]. These materials are renewable, biodegradable, and biocompatible, providing significant environmental benefits [20]. Research shows that raw material selection significantly affects the morphology and properties of nanocellulose [3,17]. This section categorizes nanocellulose sources into six main groups: wood, non-wood plants, agricultural waste, bacteria, algae, and animals. Table 1 provides detailed sources for nanocellulose production.

**Table 1:** Summary of nanocellulose forms.

Sources Group	Source	Type	Synthesis method	Morphology	Aspect	Ref.
				Dimension		
Wood	*Styphnolobium japonicum*	CNF	Chemical method with biological enzyme method 1(CM1-CPM)	Diameter: 88.2 nm;	A flat shape and damaged fiber surface	[21]
			Biological enzyme method 2 with biological enzyme method 1(BM2-CPM)	Diameter: 60.5 nm		
	*Cryptomeria fortunei*		(CM1-CPM)	Diameter: 62.6 nm	Tightly structured and interwoven networks	
			(BM2-CPM)	Diameter: 31.6 nm		
	*Pinus yunnanensis*		(CM1-CPM)	Diameter: 142.2 nm	Long columnar, sparse grid, uniform size	
			(BM2-CPM)	Diameter: 81.1 nm		
	Hemlock, Douglas fir, Cedar		TEMPO oxidation combined physical aqueous counter collision (ACC) methods	Diameter: 18.4-22 nm; Length: 815-992 nm	Uniformity of size and high transparency	[22]
	Aspen, Poplar			Diameter: 15.1-17.5 nm; Length: 652-850 nm		
	Recycled bleached pulp		Mechanical refining and high-pressure homogenization	Diameter: 19-34 nm; Aspect ratio: 92-97	Partially fibrillated during recycling; Presence of shear and fibrosis	[23]
	*Larix gmelinii*		Carboxymethylation combined with homogenization	Diameter: 3.4-3.53 nm; Length: 383.3-1210.6 nm	Average distribution of diameters; Length decreases with increasing carboxylic acid group content	[24]
	Paulownia		TEMPO oxidation combined with mild shearing	Diameter: 5 ± 3 nm; Length: 400 ± 200 nm	Narrower diameter distribution; Single or bundled basic fibers with uniform morphology	[25]
	Eucalyptus		Enzymatic hydrolysis combined with grinding method	Diameter: 8.6 ± 3.6 nm; Length: 0.76 ± 0.38 μm	Uniform fibers distribution; Rod-like or long rice-like morphology	[26]
	Eucalyptus	CNC	Sulfuric acid hydrolysis	Diameter: 4-30 nm; Length: 35-350 nm	Fusiform or twisted band structure	[27]
Non-wood plant	Licorice residues	CNF	Enzymatic pretreatment combined TEMPO-mediated oxidation method	Diameter: 130-415 nm; Aspect ratio: 54.0-62.2	Flexible morphology, pronounced lattice structure, stretched rod structure	[28]
	Honey pomelo peel		Compound enzyme method	Diameter: 30-45 nm; Length: 250-900 nm	Porous structure; Formation of lattice structure after enzymatic hydrolysis	[29]
	Apple pomace		High intensity ultrasonic treatment	Diameter: 2.59 nm; Length: several nanometers	Slender and thinner	[30]
	Carrot pomace			Diameter: 2.93 nm; Length: <1 μm	Wide and short, curved and ringed structures, presence of rice grain shaped structures	
	Rose stems		Bleaching with acid hydrolysis	Diameter: 30 ± 10 nm; Length: 420 ± 140 nm; Aspect ratio: 18	Decolourised, individually homogeneous but with low crystallinity	[31]
	Miscanthus x. Giganteus		Ammonium persulfate oxidation followed by ultrasonication	Diameter: 3.8 ± 0.8 nm; Length: 880 ± 300 nm	Fibrous morphology with high aspect ratio	[32]
	Pineapple peel	CNC	Alkali treatment combined with Sulfuric acid hydrolysis	Diameter: 15 ± 5 nm; Length: 189 ± 23 nm	Ordered and homogeneous fiber surface; Needle-like structure	[33]
	Tomato peel		Sulfuric acid hydrolysis	Diameter: 7.2 ± 1.8 nm; Length: 135-50 nm; Thicknesses: 7.2 ± 1.0 nm	Rod-like images; Flat spindle shape	[34]
	Cotton		Sulfuric acid hydrolysis	Diameter: 11.2 ± 3 nm; Length: 198.4 ± 62.8 nm	Tangle-free rod-like morphology	[35]
Agricultural waste	Sugarcane bagasse	CNF	Recombinant enzymes combined with ultrasonic treatment	Diameter: 24.7 ± 3 nm; Length: 1.3 ± 0.9 μm	Very high aspect ratio; Stable crystalline structure	[36]
			TEMPO oxidation combined with ultrasonic treatment	Diameter: 3 ± 1 nm; Length: 400 ± 200 nm	Thin fibers with a small diameter range	
		CNC	Sulfuric acid hydrolysis	Diameter: 4.36 ± 0.96 nm; Length: 127.13 ± 26.21 nm; Aspect ratio: 29.16	Rod-like and needle shapes; Spherical lignin dispersed around cellulose nanoparticles	[37]
	Coconut husk and shell	CNF	TEMPO oxidation	Diameter: 72.1-154.0 nm; Length: 108-240.2 nm	Complex spidery geometry, porous structure	[38]
	Corn husk		High pressure homogenization with TEMPO oxidation	Diameter: 15-25 nm; Length: 150 ± 50 nm	Fusiform granules, uniformly dispersed	[39]
	Peanut shell	CNC	Alkali combined with ultrasonic treatment	Diameter: 48.67 nm	Smaller diameter spherical particles	[40]
Algae	Sargasso	CNF	Alkali and acid treatment	Diameter: 3-5 nm; Length: 15-20 μm	Very thin and long fibers, few wrapped structures	[41]
	Gelium elegans, Chlorella sorokiniana			Diameter: 21.8 ± 11.1 nm; Length: 547.3 ± 23.7 nm	Fibers uniform, elongated and homogeneous	[42]
Bacteria	Acetobacter xylinum	BNC	Symbiotic interplay of microorganisms	Diameter: 20-100 nm	Tightly intertwined in random directions; porous morphology	[43]
	Acetobacter xylinum		Static fermentation combined with homogenization	Diameter: 53.24 ± 27.05 nm	High specific surface area; Reticular structure	[44]
	Komagataeibacter, Gluconacetobacter BCA263, Gluconacetobacter P1		Static culture	Diameter: 29.13-40.99 nm	Dense grid structure	[45]
			Agitated culture	Diameter: 29.51-35.64 nm	Loose and porous structure with larger pores	
	Komagataeibacter, Gluconobacter		Static culture	Diameter: 100 nm; Length: 2 μm	Banded microfiber structure, small Network nanofiber balls	[46]
Animal	Tunicates	CNF	Sulfuric acid hydrolysis combined with ultrasonic treatment	Diameter: 15.3-21.9 nm; Length: 1150-1440 nm	Nanolaminar cross-section and shorter, straighter fiber structure	[47]
			TEMPO oxidation combined with ultrasonic treatment	Diameter: 15.5 ± 1.3 nm; Length: 1590 ± 1010 nm	Decomposed individual microfibers and rough bundle structure	
		CNC	Sulfuric acid hydrolysis	Diameter: 16.6 nm; Length: several micrometers	Rod-shaped form, porous structure	[48]

#### 2.1.1 Wood-based sources

Wood, a widely available and renewable natural composite, primarily consists of cellulose, hemicellulose, and lignin. Renowned for its strength, stiffness, toughness, and low density, wood's abundant cellulose content and availability make it an excellent raw material for industrial nanocellulose production [[48], [49], [50]]. Nanocellulose from wood is typically divided into hardwood and softwood. Hardwoods include species such as Eucalyptus, Birch, Poplar, while softwoods comprise Pine, Douglas fir, and others. While hardwoods generally possess shorter fibers and lower lignin content than softwoods, their dense microstructure may require more intensive physical treatment, such as homogenization, to achieve effective fibrillation [51]. In contrast, softwoods undergo fibrilization more efficiently, with lower energy consumption, making them more suitable for commercial applications [52]. Studies indicate that renewable wood biomass for nanocellulose extraction provides notable benefits, including improved production efficiency, reduced energy use, lower environmental impact, and enhanced safety [53]. Growing interest in lignocellulose-based bio-nanocomposites highlights the potential of lignocellulosic nanocellulose for biomedical applications, marking a promising path for future research [54].

However, the purity and biomedical suitability of lignocellulose-derived nanocellulose largely depend on the efficiency of delignification and purification. Well-purified nanocellulose forms a uniform nanofibrillar network, provides reliable mechanical reinforcement, and enables controllable porosity. In contrast, insufficient purification can leave residual non-cellulosic components, which may compromise surface reactivity, membrane uniformity, and long-term biocompatibility [55].

#### 2.1.2 Non-wood plant sources

Compared with wood, non-wood plants, which contain lower lignin content, are valuable sources for nanocellulose production. They can facilitate the formation of nanocellulose with smaller diameters, higher aspect ratios and more open fibrillar networks. Such structural features improve chain entanglement, water uptake and interfacial contact within the membrane matrix, thereby enhancing cell adhesion, nutrient transport and flexible mechanical performance in biomedical applications [56]. This category encompasses bast fibers such as ramie, jute, kenaf, flax, and hemp, along with herbaceous plants, root vegetables, and succulents [17,54]. Notable shrub sources include cotton and hibiscus [55,56]. Other plant sources include reeds [57], bamboo [58], sugarcane [36], grasses such as *Miscanthus giganteus* [32] and Imperato brasiliensis [59], corn husks, apples [30], carrots [30], and agave [60]. These plants exhibit properties advantageous for nanocellulose development, including cost-effectiveness, reduced equipment wear, high specific strength, stiffness, and low density. These attributes strongly support the use of non-wood plant fibers for nanocellulose and composite production, with their supply chains becoming increasingly robust.

#### 2.1.3 Agricultural waste sources

Agricultural and forestry activities are widespread globally, generating agricultural waste that can serve as a potential raw material for nanocellulose production, such as crop residues [36,39,58,59], fruit peels [32,60], and other plant-based waste products [37,61]. However, these feedstocks are compositionally more complex than conventional plant-derived cellulose. Such heterogeneity can affect fibrillation behavior, and insufficient purification may result in broader size distributions and reduced structural uniformity of the resulting membranes. Studies have shown that mild mechanical disintegration, alkaline treatment and bleaching can promote fiber swelling and facilitate improved network formation, thereby optimizing the morphological characteristics of waste-derived nanocellulose [62].

Utilizing agricultural waste offers clear economic and environmental advantages. Nanocellulose derived from these sources features a large surface area, nanoscale dimensions, high crystallinity, and high hardness, along with being inherently biodegradable and renewable [63]. These characteristics make it suitable for the sustainable fabrication of scaffold membranes. However, stricter control is still required to ensure consistent product quality and standardization.

#### 2.1.4 Bacterial sources

Bacterial nanocellulose (BNC) is a high-molecular-weight polysaccharide produced extracellularly by Gram-negative bacteria, with Acetobacter xylinum being one of the most efficient producers [64]. BNC shares the same chemical properties as plant cellulose but lacks by-products such as lignin, pectin, and hemicellulose. It forms a unique network of fine fibers [65]. BNC typically exhibits higher purity, greater polymerization, and higher crystallinity compared to plant-derived cellulose, contributing to its superior mechanical strength and stability [66]. BNC has spurred the development of biomedical products due to its excellent biocompatibility, with applications in tissue scaffolds, artificial blood vessels, and artificial skin [67]. However, the complex production process and low efficiency hinder the industrial-scale availability of BNC at low cost. Although strategies like cell-free technology [68], bioreactors [69], and surface functionalization have been employed to enhance BNC yield and properties [70], further research into culture media is needed to determine whether BNC can become the most cost-effective and efficient source of nanocellulose in the future [71].

#### 2.1.5 Algal sources

Algae, such as Cladophora and Cystoseria myrica, are recognized as important sources of nanocellulose with environmental benefits [72]. The advantages of algae-derived cellulose over lignocellulose include: (i) rapid growth and short production cycles of marine algae, which can meet raw material demands for nanocellulose production [73]; (ii) algae have minimal or no natural physical and chemical barriers, eliminating the need for extensive chemical treatment and improving cellulose accessibility [74]; (iii) marine algae yield high carbohydrates and efficiently use carbon dioxide, light, and inorganic nutrients to grow under simple conditions without incurring significant costs [75]. Current research shows that algae-derived nanocellulose has high crystallinity and enhanced thermal stability, making it a valuable reinforcing agent for nanocomposites [76]. Nanocellulose from Cladophora has been evaluated for potential pharmaceutical and biomedical applications, with future research in this area showing significant potential [77].

#### 2.1.6 Animal sources

Animal-derived nanocellulose primarily comes from tunicates (Chordata), with sea squirts being the most notable example. These marine invertebrates are filter feeders and typically yield long fibrillar structures [78]. Such architectures can effectively reinforce otherwise fragile biomedical membranes. Song et al. [[75], [76], [77]] developed a regenerated nanocellulose membrane from sea squirts and demonstrated its non-toxicity and excellent biocompatibility in skin wound healing applications. Moon et al. [47] prepared a nanocellulose film from sea squirts, which exhibited outstanding mechanical properties and thermal conductivity, making it suitable for use in flexible electronic devices. However, nanocellulose derived from tunicates remains less competitive than plant-based sources in terms of feedstock availability, processing cost, and scalability. As a result, its practical use is more suited to the fabrication of specialized, high-performance biomedical materials rather than large-scale industrial production [79].

Overall, nanocellulose can be derived from a wide range of sources, which not only influence fibrillar morphology but also determine purity, impurity content, biocompatibility, and economic feasibility [80]. Table 2 provides a qualitative comparison of nanocellulose from different sources for biomedical applications. At present, plant-derived nanocellulose remains the primary choice for large-scale industrial production. Waste-derived nanocellulose offers greater economic and environmental benefits but requires stricter control over source heterogeneity. Bacterial nanocellulose exhibits the highest purity and can form intrinsically entangled water–nanofiber networks; however, its relatively high production cost and low yield remain key barriers to clinical translation. Algal and tunicate-derived nanocellulose represent emerging alternatives, with potential for high-performance, small-batch biomedical applications.

**Table 2:** Qualitative comparison of nanocellulose from different sources for biomedical applications.

Source category	Typical examples	Purity	Impurities	Biocompatibility	Cost	Scalability	Ref.
Wood-based sources	Hard wood, softwood	★★★	Lignin, hemicellulose	★★★	★★	★★★★	[53,55,81]
Non-wood based sources	Cotton, bamboo, miscanthus, flax	★★	Lignin, hemicellulose, pectin	★★★	★	★★★★	[56,82]
Waste-derived sources	Food waste, wheat straw, bagasse, husk	★	Lignin, hemicellulose, residual organics, contaminants	★★	★	★★★	[[61], [62], [63]]
Bacterial sources	Acetobacter xylinum	★★★★★	Culture medium residues	★★★★★	★★★★	★★	[66,71,83]
Algal sources	Sargasso	★★★	Non-cellulosic polysaccharides, proteins, minerals	★★★★	★★★	★★	[72,76,77]
Marine animal sources	Tunicate	★★★	Proteins, minerals, salts	★★★★	★★★★★	★	[78,84,85]

Note: The “★” symbols in the table indicate the level of a characteristic; the more “★” symbols there are, the higher or stronger that characteristic is.

### 2.2 Types of nanocellulose

#### 2.2.1 Original nanocellulose

**Fig. 3:**
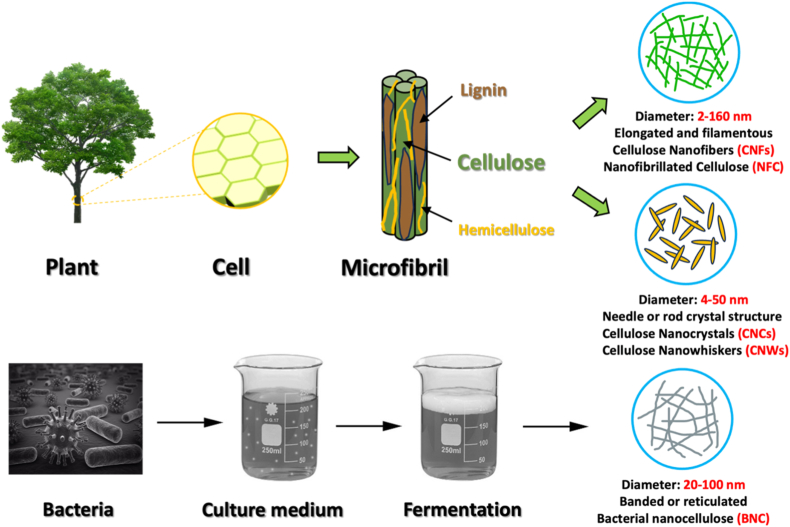
Summary of original nanocellulose types.

Nanocellulose is typically classified into three types depending on its source and production method: (i) cellulose nanocrystals (CNCs), also known as nanocrystalline cellulose (NCC), cellulose nanowhiskers (CNWs), and rod-like cellulose microcrystals; (ii) cellulose nanofibers (CNFs), also known as nanofibrillated cellulose (NFC) and cellulose nanofibrils; and (iii) BNC, also referred to as microbial cellulose [3,86]. Fig. 3 shows the different types of nanocellulose.

##### 2.2.1.1 Cellulose nanocrystals (CNCs)

CNCs (also known as CNWs in early research) are rod-like short fibers that match the size of isolated cellulose crystals, typically measuring 4 to 50 nm in diameter and 100 nm to several micrometers in length [3,84]. CNCs can be extracted from various cellulose-rich biomass sources, including wood, cotton, hemp, flax, and tunicates. The crystallinity of CNCs varies depending on the source, which significantly affects the size of the isolated crystals [3]. CNCs are primarily obtained by removing the amorphous cellulose regions through strong acid hydrolysis.

CNCs can also be produced via gentle enzymatic hydrolysis, which enhances their mechanical and thermal properties [84,85]. CNCs exhibit characteristics such as a large surface area, high crystallinity, low thermal expansion coefficient, and high tensile strength [87], making them suitable for applications such as reinforcement in composites and fillers in coatings [79,80,83]. Moreover, CNCs are biocompatible and biodegradable, and CNC-based hydrogels are widely used in biomedical fields, including drug delivery, wound dressings, and tissue engineering scaffolds [88,89]. CNCs can also be applied in antibacterial and antiviral applications, biosensors, and gene delivery through surface modification [87,90,91].

##### 2.2.1.2 Cellulose nanofibrils (CNFs)

CNFs are typically longer fibers with amorphous regions and soft, elongated chains. These fibers usually have diameters ranging from 2 to 160 nm and lengths of several micrometers [3,92]. Unlike CNCs, CNFs are typically extracted from cellulose chains through mechanical processes. However, extraction efficiency can be improved by combining chemical and enzymatic methods [93]. CNFs have a higher aspect ratio, larger surface area, and more hydroxyl groups. These features facilitate property enhancement and expand their applications through surface modifications, such as TEMPO-oxidized cellulose nanofibers (TOCNs) [94] and carboxymethylated cellulose nanofibers (CM-CNFs) [95]. CNFs typically possess high tensile strength and flexibility, although their specific properties and geometric configurations are influenced by the cellulose source and preparation method [96]. CNFs can also undergo chemical modifications to improve their compatibility with other materials. Composite nanomaterials based on CNFs exhibit excellent mechanical properties, barrier functions, and biocompatibility, making them suitable for applications in civil engineering, food packaging, papermaking, and medicine [[97], [98], [99], [100]].

##### 2.2.1.3 Bacterial nanocellulose (BNC)

BNC is a highly crystalline linear glucose polymer primarily synthesized by the bacterium Ochratoxium xylinum. It typically forms twisted ribbon-like or net-like structures, with diameters ranging from 20 to 100 nm and lengths in the micron range and is characterized by a large unit surface area [101]. *G. xylinus* is the primary microbial producer of BNC and serves as a model system for studying the biosynthetic mechanism of BNC in bacteria [102]. BNC is typically produced by two methods: static culture and agitated culture [45]. Cellulose produced by agitated culture has lower mechanical strength and yield compared to that produced by static culture, although static culture requires more space and time [97,99]. Compared to CNCs and CNFs, BNC remains pure, free from lignin, hemicellulose, and pectin. This results in a higher weight-average molecular weight (M_w_), higher degree of polymerization, greater crystallinity, and superior mechanical stability [3,63]. Research has shown that using tension-assisted twisting technology, BNC can be drawn into a compact bundle structure to significantly enhance its mechanical strength [83]. Additionally, BNC has become one of the most prominent materials in the biomedical field due to its excellent biocompatibility [65].

#### 2.2.2 Regenerated nanocellulose

Regenerated cellulose is a fiber product obtained through the physical and chemical treatment of cellulose-rich materials such as cotton linter pulp, wood pulp, bamboo pulp, and other cellulose sources, followed by wet spinning to form a concentrated cellulose solution or its derivatives. After dissolution and regeneration, the crystalline structure of cellulose typically transforms from cellulose I to cellulose II, as the molecular chains rearrange from a parallel to an antiparallel orientation. However, this transformation is not always complete, and regenerated cellulose often contains both cellulose II crystalline regions and amorphous domains, depending on factors such as the solvent system, regeneration conditions, cellulose source and degree of polymerization.

The dissolution difficulty of cellulose, arising from its long molecular chains interconnected by densely packed hydrogen bonds, has long been a critical challenge for its use as a polymeric material component [103]. During the regeneration process, the hydrogen-bonding network and chain packing are rearranged. Density functional theory (DFT) studies have demonstrated that cellulose I (including cellulose Iα and Iβ) chain-sheet models exhibit right-handed twists, whereas cellulose II (010) and (020) models twist in the opposite direction, displaying right- and left-handed chirality, respectively [104]. This structural reconfiguration alters surface polarity, crystallinity, and flexibility. Such characteristics endow regenerated cellulose with unique luster, excellent hygroscopicity, breathability, and antistatic properties, making it highly attractive to consumers. Furthermore, the modified hydrogen-bond network enhances the solubility compatibility and spinnability of regenerated cellulose within polymer–solvent systems, thereby facilitating electrospinning and the formation of composite membranes [103]. It is worth noting that regenerated cellulose is not strictly speaking a type of nanocellulose, as its dimensions are not typically in the nanoscale range. However, through further customized processing to break it down, regenerated nanocellulose can be formed. This type of nanomaterial derived from ‘regeneration’ not only retains the processing scale and environmental friendliness of regenerated materials but also offers the performance advantages of the nanoscale and is therefore also classified as a new type of nanocellulose.

**Fig. 4:**
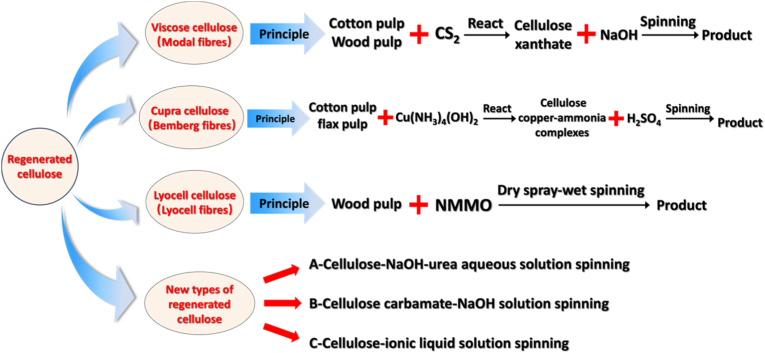
Summary of regenerated cellulose types.

Efforts have focused on producing industrial-scale, non-derivatized, and environmentally friendly regenerated cellulose, such as viscose, cupra, and lyocell cellulose. Additionally, the development of new cellulose solvents has facilitated the advancement of regenerated cellulose fibers, including alkali/urea aqueous solutions, ionic liquids, and cellulose carbamate systems [100]. Fig. 4 summarizes the various types of regenerated cellulose.

##### 2.2.2.1 Viscose cellulose/fibres

Cellulose xanthate, produced by reacting cotton or wood pulp with CS_2_ after treatment with NaOH, is soluble in water or dilute NaOH solution, forming a yellow cellulose viscose solution. The solution is then spun, followed by coagulation and regeneration in (NH_4_)_2_SO_4_ and dilute sulfuric acid solutions. After washing and drying, white cellulose fibers, known as viscose fibers, are obtained [105].

Modal⑧ is a viscose fiber with high wet modulus, primarily made from spruce and beech pulp, and processed using an improved spinning method. The production process involves using a high esterification, high-viscosity solution and spinning in a multi-component, low-concentration, low-temperature solidification bath. This overcomes the limitations of ordinary viscose fibers, such as low wet strength and poor dimensional stability.

However, the viscose process is complex, with a long production cycle. It also releases toxic CS_2_ and H_2_S gases, which harm human health, pollute the environment, and disrupt the ecological balance [106]. Therefore, the viscose industry is undergoing a shift from traditional low-tech, single-species products to high value-added, differentiated, and diversified offerings.

##### 2.2.2.2 Cupra cellulose/fibre

Cotton and flax cellulose can be dissolved in cupro solution at room temperature. Cellulose was regenerated from the cupro solution using a dilute sulfuric acid aqueous solution, and cupro fibers were successfully prepared after drying. Bemberg⑧ is a typical product made from cupro cellulose. The monofilament of cupro fibers is very thin, with a rounded cross-section and no core structure [105]. Consequently, fabrics made from cupro are soft to the touch and possess a luster similar to silk, rather than viscose. Additionally, the hygroscopicity of cupro fibers is similar to that of viscose fibers. Under the same dyeing conditions, cupro fibers have a higher dye affinity and a darker color compared to viscose fibers. The dry strength of cupro fibers is similar to that of viscose fibers, but their wet strength and abrasion resistance are superior [107]. From an environmental perspective, the cupro fiber production process requires treatment of copper, waste acid, and wastewater generated during production.

##### 2.2.2.3 Lyocell cellulose/fibre

Lyocell fiber is a new generation of regenerated cellulose fiber produced by directly dissolving wood pulp in an aqueous solution of N-methylmorpholine-N-oxide (NMMO) followed by dry-jet wet spinning. The NMMO process differs from the viscose process as it does not involve chemical reactions in the preparation of regenerated cellulose fibers [108]. Compared to viscose fibers, lyocell fibers have high strength, wet modulus, and dimensional stability. They can also be blended with fibers such as linen, cashmere, and wool, earning them the title “green fibers of the 21st century” [109].

However, several issues remain in the Lyocell fiber process that need addressing [105,106]: 1) NMMO decomposes at high temperatures; 2) chromophore by-products from the dissolution process contaminate the finished fiber; 3) cellulose degradation during spinning compromises fiber mechanical properties and causes fluctuations in production; 4) fibrillation of Lyocell fibers; 5) stabilizer additives lead to additional side reactions; and 6) dissolution and exothermic reactions pose explosion risks, threatening production safety.

##### 2.2.2.4 New types of regenerated cellulose/fibre

New regenerated cellulose is emerging as an environmentally friendly, high-performance alternative to traditional fibers. These advances highlight the growing pursuit of sustainable and innovative materials, positioning regenerated cellulose as a key component of the future textile and biomedical sectors. Alkali/urea aqueous systems (e.g., 7% NaOH/12% urea or 4.6% LiOH/15% urea) can rapidly dissolve cellulose within 2 min through low-temperature-induced hydrogen bonding between solvent molecules and cellulose chains [105,106]. This enables fast preparation of concentrated cellulose solutions and direct fiber production using existing viscose spinning equipment, yielding fibers with mechanical properties comparable to viscose [107,108]. This technology utilizes existing equipment, minimizing initial investment. Furthermore, alkali/urea aqueous solvent systems do not release toxic chemicals, making them particularly suitable for “clean” industrial production.

Derivatives of cellulose reacted with urea are soluble in dilute alkalis and can be regenerated in acids to produce fibers or membranes. A method for reacting cellulose with urea to form cellulose carbamate has been developed [103]. Cellulose carbamate can be dissolved in a NaOH aqueous solution at a specific concentration to produce spinning liquid. Since carbamate is easily hydrolyzed in alkaline media, it must be dissolved and stored at temperatures below 0 °C. The resulting Carbacell fibers, regenerated in sulfuric acid or sodium carbonate, have uniform ovoid cross-sections similar to Lyocell fibers [110]. However, cellulose carbamate synthesis remains energy-intensive, and solid- or liquid-phase processes are inefficient or solvent-consuming.

Ionic liquid-based regenerated cellulose is another class of regenerated cellulose. The spinning process for cellulose dissolved in ionic liquids is like that of Lyocell fibers, with options for wet spinning or dry-jet wet spinning. Regenerated cellulose fibers from cellulose dissolved in some ionic liquids exhibit mechanical properties similar to or slightly superior to those of Lyocell fibers. They outperform viscose fibers produced by the traditional viscose process [[111], [112], [113], [114], [115]]. However, high ionic liquid cost and recycling difficulties hinder large-scale commercialization, especially as Lyocell production expands.

## 3 Pretreatment and extraction process for nanocellulose

As discussed in the previous section, different nanocellulose sources and types show clear differences in chemical composition, fiber structure, purity, yield, and processing difficulty. These source-dependent features directly affect the choice of pretreatment and extraction methods. They also influence the type, morphology, surface chemistry, and biocompatibility of the final nanocellulose products. The production of nanocellulose involves two stages: (1) pretreatment of lignocellulosic materials to isolate cellulose fibers, and (2) decomposition of these fibers into nanoscale dimensions [116]. The production of nanocellulose from raw materials employs various pretreatment and extraction methods, which critically affect the properties of the final product [63]. For nanocellulose products used in biomedical applications, the cell compatibility and biotoxicity of chemically processed CNC are often limited by residual acid and base groups. Enzyme-pretreated mechanically processed CNF avoids irritating chemicals and demonstrates superior biocompatibility. On the other hand, the industrial production and application of nanocellulose face significant challenges. Cost and environmental issues have always been key factors to consider in the large-scale production of nanocellulose products in high-end industries. The energy consumption and maintenance costs of physical production greatly limit the commercial application of disposable medical products, while chemical production requires consideration of the problems caused by solvent recovery and environmental safety. Consequently, the cost-effectiveness of nanocellulose scaffold membranes must be balanced with yield, sustainability, and scalability. A thorough understanding of the latest advances in nanocellulose processing technology can provide a foundation for selecting the optimal preparation method for nanocellulose scaffold membranes. Researchers are working to develop production methods that are more environmentally friendly, cost-effective, and scalable [115,116].

### 3.1 Pretreatment for nanocellulose

**Fig. 5:**
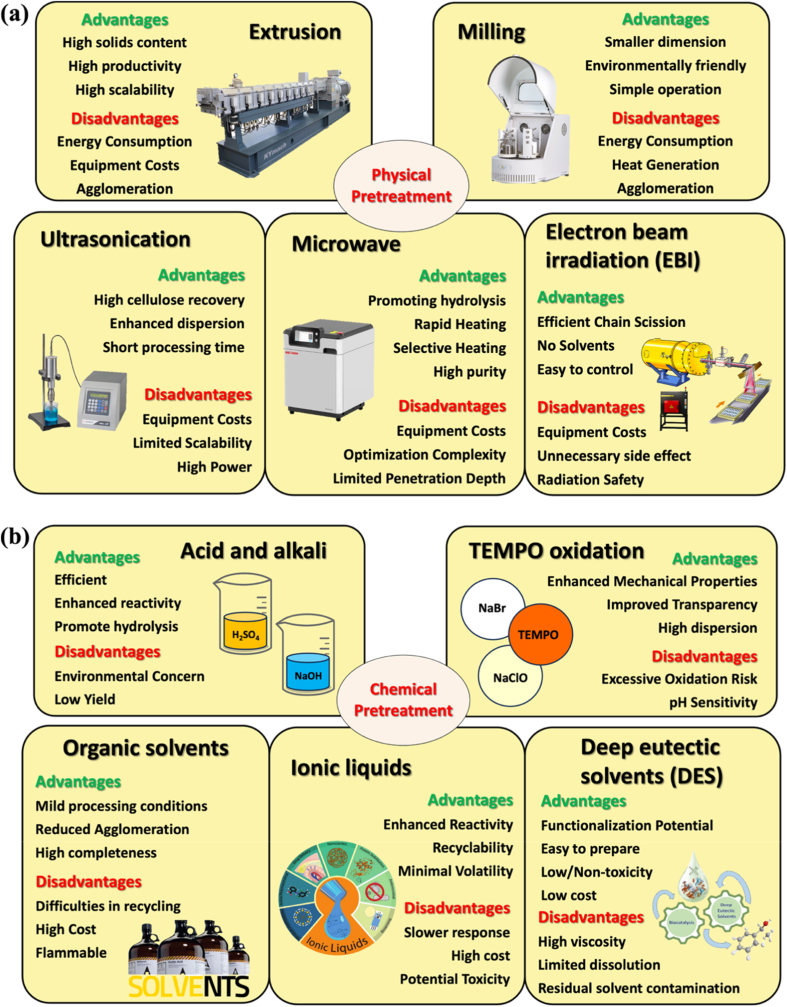

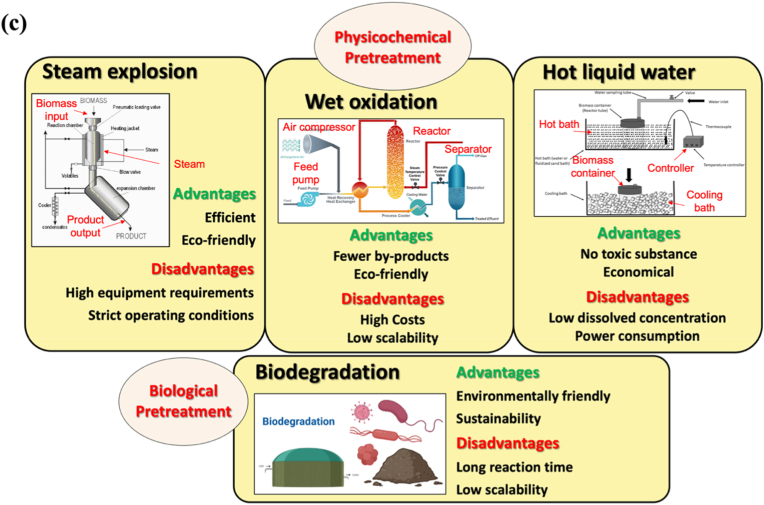
Summary of pretreatments for nanocellulose (a) Physical pretreatments; (b) Chemical pretreatments; (c) Physicochemical and biological pretreatments.

Pretreatment technologies target lignocellulosic biomass by removing lignin, decomposing hemicellulose, and enabling subsequent cellulose processing [117]. Pretreatment also breaks cellulose hydrogen bonds, modifies the crystallinity of microfibrils, increases hydroxyl group availability, and enhances cellulose reactivity [118]. Consequently, pretreatment is not only a purification step but also a critical structural regulation process, significantly influencing the type, dimensions, and interfacial properties of the resulting nanocellulose. Pretreatment methods are broadly classified into physical, chemical, physicochemical, and biological approaches [119]. The following sections review both traditional and emerging technologies for pretreatment. Fig. 5 illustrates various pretreatment techniques for nanocellulose.

(Adapt from Ref. [120] with permission. Copy right Taylor & Francis, 2024)

#### 3.1.1 Physical pretreatment

##### 3.1.1.1 Extrusion pretreatment

Extrusion is a widely used thermophysical pretreatment technique that disrupts the rigid structure of wood cellulose, facilitating carbohydrate utilization through enzymatic hydrolysis [121]. Extrusion machines are classified into single-screw extruders, which use a single rotating screw, and twin-screw extruders, which consist of multiple screw elements in a cylindrical configuration [122]. Research indicates that nanofibers produced via extrusion generally exhibit higher efficiency, yield, and solid content. Ho et al. [123] studied the impact of twin-screw extruders on pulp fibers, achieving a fibrous solid content of up to 50%. Khadija Trigui et al. [124] devised a method to produce nanocellulose hydrogels using twin-screw extruders, achieving enhanced production and pumping efficiency. Lu et al. [125] produced lignocellulose nanofibrils from bamboo and other raw materials using screw extrusion, achieving yields over 70%. The resulting nanocellulose films exhibited excellent tensile properties. However, high energy consumption and operating costs pose challenges to the large-scale production of nanocellulose.

##### 3.1.1.2 Milling pretreatment

Milling employs mechanical force to disrupt cellulose microstructure, transforming it into nanoscale fibers or crystals [119]. Common grinding methods include wet disk grinding, dry grinding, ball milling, hammer milling, rod milling, and vibratory grinding. These techniques offer advantages such as particle size reduction, decreased cellulose crystallinity, and enhanced process efficiency [126]. Chen et al. [127] developed a method for preprocessing cotton fiber nanocellulose via wet grinding, yielding CNF with widths of 10–30 nm and high aspect ratios. Lee and Sudhagar Mani [128] observed that wet grinding's high energy and water demands increase energy consumption during CNF production. To address this, they used dry grinding to prepare CNF with an average diameter of 119 nm, which also prevented process clogging. Frederikus Tunjung Seta et al. [129] employed ball milling to preprocess bamboo-derived CNCs. The technique reduced fiber size, facilitating subsequent acid hydrolysis and yielding CNC particles with lengths of 105.6–223.8 nm. However, high energy consumption and incomplete lignin removal hinder enzymatic hydrolysis of cellulose and hemicellulose, representing two major drawbacks of this process [130]. Studies indicate that grinding pretreatment energy requirements depend on the process and biomass feedstock type. Therefore, choosing the appropriate grinding method for specific biomass materials is vital for optimizing yield and energy efficiency [125,126].

##### 3.1.1.3 Ultrasonication-based pretreatment

Ultrasonic pretreatment is an eco-friendly and efficient technique targeting non-crystalline cellulose regions through cavitation effects, which disrupt hydrogen bonds in cellulose. Cavitation bubbles formed during the process disrupt cellulose and hemicellulose, enhancing the accessibility of acids and cellulase during hydrolysis [115,127]. This method is simple to operate, requires mild conditions, and minimizes the need for chemical reagents. Ultrasonic pretreatment of lignocellulosic biomass often achieves high cellulose recovery and effective lignin removal [130]. In practice, ultrasonic pretreatment is often combined with enzymatic and chemical treatments to improve nanocellulose yield and quality. Wang et al. [131] demonstrated that ultrasonic pretreatment acts on multiple targets, enhancing subsequent enzymatic reactions in lignocellulose. Govindarajan Ramadoss and Karuppan Muthukumar [132] utilized ultrasonic-assisted ammonia pretreatment on sugarcane bagasse for sugar fermentation, achieving 95.78% cellulose recovery and 58.14% delignification. Tang et al. [133] employed low-intensity ultrasonic-assisted sulfuric acid hydrolysis to extract CNC, increasing its yield from 33.0% to 40.4% and achieving 90.38% crystallinity. Excessive ultrasonic power may hinder the pretreatment process by causing bubble formation near the ultrasonic transducer, which disrupts efficient energy transfer to the liquid medium [134]. Future research should prioritize understanding the mechanisms of ultrasonic enhancement in chemical and enzymatic systems to optimize processes and advance high-quality nanocellulose production [135].

##### 3.1.1.4 Microwave-based pretreatment

Microwave pretreatment is an advanced heat treatment technique utilizing electromagnetic fields, where microwave radiation penetrates cellulose materials and induces rapid rotation of polar molecules like water. The rapid molecular rotation in the microwave field generates frictional heat, ensuring uniform material heating [136]. This method rapidly heats cellulose, breaking hydrogen bonds between molecular chains and facilitating subsequent nanocrystallization [120]. Compared to traditional thermophysical methods, microwave pretreatment provides faster heat transfer, shorter reaction times, selective heating, and higher energy efficiency [131,137]. Microwave pretreatment is frequently combined with other methods to improve nanocellulose yield and production efficiency. Paulo H. Camani et al. [138] showed that microwave-assisted pretreatment removed non-cellulosic compounds from eucalyptus waste, achieving 99.1% cellulose purity. Qian et al. [139] developed a microwave-assisted dilute acid pretreatment method, which promoted enzymatic hydrolysis, yielding CNCs with 84.4% efficiency and improved crystallinity and thermal stability over commercial nanocellulose. Wang et al. [140] reported that microwave-induced structural changes in bamboo reduced reaction time and chemical consumption during oxalic acid hydrolysis, enhancing nanocellulose production. However, the high cost of microwave pretreatment equipment and the need for strict reaction control limit its widespread industrial application. Future research should prioritize developing cost-effective and efficient microwave pretreatment equipment and processes for large-scale nanocellulose production.

##### 3.1.1.5 Electron beam irradiation (EBI) pretreatment

Electron beam irradiation (EBI) is an emerging nanocellulose technology used for separating cellulose fibers from wood cellulose and reducing their size to the nanoscale (fibrosis) [116]. This technology employs accelerated electron beams to irradiate wood cellulose, initiating free radical formation, cross-linking, chain scission, and decrystallization to disrupt its internal structure [141]. EBI pretreatment offers several advantages, such as environmental friendliness, efficiency, and ease of control [120]. Lee et al. [142] combined EBI pretreatment with high-pressure homogenization to produce CNCs (23–31 nm width; 128–747 nm length) with stable dispersibility and high transparency in dispersions. Wu et al. [143] extracted nanocellulose from waste cotton fabrics, noting that EBI reduced cellulose polymerization and increased crystallinity. The nanocellulose exhibited high thermal stability and excellent emulsifying properties. Additionally, EBI radiation doses significantly influence the modification of nanocellulose. Zhang et al. [144] prepared carboxymethylated nanocellulose (5–40 nm diameter; 50–300 nm length) via EBI pretreatment and found that higher irradiation doses enhanced its modification. However, EBI technology faces drawbacks, including high equipment costs, significant cellulose consumption, and potential adverse chemical reactions. Research on nanocellulose production via EBI pretreatment remains limited, requiring further studies to elucidate its impact on nanocellulose properties. In the future, EBI could be integrated with physical, chemical, or enzymatic methods to develop hybrid processes for nanocellulose production [116].

Overall, physical pretreatment methods preserve the intrinsic chemical structure of nanocellulose while improving fibrillation efficiency, which is advantageous for biomedical applications requiring high cytocompatibility and minimal chemical residues [145]. However, their major limitation remains high energy consumption, which significantly affects cost and scalability. As a result, physical pretreatments are often combined with mild chemical or enzymatic steps to reduce fiber recalcitrance prior to fibrillation. Ongoing efforts are focused on developing integrated strategies to minimize cumulative yield losses during cellulose purification and bleaching processes [144,146].

#### 3.1.2 Chemical pretreatment

##### 3.1.2.1 Acid pretreatment

Acid pretreatment is a critical step in converting lignocellulosic biomass. It uses acidic reagents, such as sulfuric, acetic, or phosphoric acid, to remove lignin and hemicellulose, enhancing enzyme accessibility to cellulose [130]. This method is particularly effective for producing biofuels, such as bioethanol [147]. Both dilute and concentrated acids are applicable for processing lignocellulose. However, concentrated acids pose higher corrosion risks, making them less preferable [148]. Dilute acid hydrolysis is widely used due to its low acid consumption. Biomass pretreatment with dilute acid can achieve high sugar and ethanol yields [140,142]. Although acid pretreatment enhances enzymatic digestibility and saccharification rates, it has drawbacks, including high recovery costs and the formation of inhibitory compounds. Innovative processes, such as solid catalysts, offer alternative approaches to improving process performance [149].

##### 3.1.2.2 Alkali pretreatment

Alkaline pretreatment is particularly effective for agricultural residues and herbaceous crops with low lignin content. Alkaline treatment breaks bonds between lignin and carbohydrate polymers and partially dissolves lignin, enhancing enzymatic hydrolysis and biofuel production [150]. Common alkaline agents include sodium hydroxide, calcium hydroxide, potassium hydroxide, and ammonia [130]. Alkaline pretreatment operates at lower temperatures and pressures compared to acidic hydrolysis. It produces fewer fermentation inhibitors, involves simpler preparation, and is suitable for industrial applications. Chen et al. [151] applied alkaline pretreatment to bamboo stalks to produce regenerated lignocellulose. They observed a 128.3% improvement in the tensile strength of regenerated fibers compared to untreated fibers and noted an increase in the viscosity of the spinning solution. The main disadvantages of alkaline pretreatment include high chemical costs, lengthy reaction times, and its ineffectiveness for biomass materials with high lignin content, such as wood [152].

##### 3.1.2.3 TEMPO-mediated oxidation pretreatment

2,2,6,6-tetramethylpiperidine-1-oxyl (TEMPO) oxidation is a commonly used chemical pretreatment for nanocellulose, typically conducted with sodium hypochlorite (NaClO) and sodium bromide (NaBr) as co-reagents. Compared to acid-base treatment, TEMPO-pretreated nanocellulose exhibits higher carboxyl content, which enhances dispersibility and stability in aqueous solutions. The mild reaction conditions make this method environmentally friendly; however, its cost is relatively high [120]. Barbash et al. [153] extracted nanocellulose from reed stems via the TEMPO oxidation method. The resulting membranes exhibited a density of 1.51 g/cm^3^, 82.4% transparency, and a tensile strength of 69.7 MPa. Both the amount of TEMPO and the oxidation time significantly influenced product performance.

##### 3.1.2.4 Organic solvent pretreatment

Organic solvent pretreatment is another method for producing pure cellulose by decomposing hemicellulose and lignin under heat. Common organic solvents include ethanol, acetone, and organic acids [154]. Compared to traditional methods, organic solvent pretreatment is more efficient at separating hemicellulose, leading to higher cellulose purity. The high-quality lignin separated can be widely used as valuable chemicals in various industrial applications [155]. Zhang and Liu [156] efficiently isolated CNC from eucalyptus hardwood using high-energy oxidation and organic solvent dissolution. This environmentally friendly method yielded a CNC suspension with an 87% yield, exhibiting good stability and optical properties. Araceli et al. [157] investigated various pretreatment methods for extracting CNC from agricultural waste. The results showed that CNC obtained via organic solvent pretreatment had smaller dimensions and higher surface charge than those from alkali treatment, making it more suitable for further chemical modification. Although organic solvent pretreatment offers advantages in cellulose purity and lignin recovery, it faces challenges related to solvent costs, recovery, and safety [155]. Improving solvent recovery technology, reducing costs, and developing more sustainable solvents are essential for making this pretreatment method feasible at an industrial scale.

##### 3.1.2.5 Ionic liquid pretreatment

Ionic liquids are considered green alternatives to volatile organic solvents. Their unique properties, such as non-volatility, selectivity, and recyclability, make them a promising approach for extracting cellulose from lignocellulosic biomass [158]. The commonly used ionic liquids in pretreatment are imidazole-based, where specific ion pairs break the hydrogen bonds in lignocellulose, disrupt its structure, and promote component separation [159]. Kazuaki et al. [160] prepared CNF from sugarcane bagasse using ionic liquid pretreatment. The resulting product exhibited a higher surface area, demonstrating that this method enhances the performance of lignocellulosic nanofibers (LCNFs) as bio-nanocomposite reinforcing agents. Currently, the cost and potential toxicity of ionic liquids remain major concerns in nanocellulose preparation. Developing cost-effective and environmentally friendly ionic liquids may offer a viable solution for large-scale biorefining and sustainable cellulose extraction.

##### 3.1.2.6 Deep eutectic solvents (DES) pretreatment

Pretreating lignocellulose with deep eutectic solvents (DES) is gaining popularity. DES is a green solvent made by mixing hydrogen bond donors (HBD) and hydrogen bond acceptors (HBA) in specific proportions, forming a eutectic mixture [161]. Compared to traditional ionic liquids, DES pretreatment of nanocellulose offers advantages such as environmental friendliness, low cost, simplicity, and non-toxicity. The high biomass solubility and conversion rate, driven by intramolecular hydrogen bonds in DES, offer greater potential for optimal nanocellulose pretreatment schemes [162]. Tan et al. [163] proposed a method to enhance microbial lipid production by pretreating wheat straw with a novel ternary deep eutectic solvent, achieving lignin removal (92.37%), cellulose recovery (89.12%), and hemicellulose removal (90.35%). The pretreated wheat straw exhibited higher sugar conversion and lipid yields during enzymatic hydrolysis and microbial fermentation. Additionally, DES-pretreated nanocellulose often exhibits higher viscosity [164]. Future research aims to optimize DES formulations and processes for large-scale cellulose extraction and commercial lignocellulose applications.

#### 3.1.3 Physicochemical pretreatment

##### 3.1.3.1 Steam explosion pretreatment

Steam explosion pretreatment is a physicochemical method for lignocellulose processing, involving short-term treatment with high-pressure steam (160-240 °C), causing explosive decomposition and breaking of glycosidic bonds in hemicellulose and cellulose, as well as cleavage between hemicellulose and lignin [116,154]. The main advantages of steam explosion pretreatment are high efficiency and low chemical requirements. The explosive decompression process disrupts the lignocellulosic matrix, making cellulose easier to separate and improving the efficiency of subsequent enzymatic or chemical treatments [122]. Dagem et al. [165] evaluated the effect of steam explosion pretreatment on CNC yield from poplar wood and found that it significantly increased both yield and thermal stability during the acid hydrolysis process. However, steam explosion is non-selective and often requires additional steps to optimize the quality and purity of the extracted nanocellulose. With further optimization, steam explosion is expected to become a scalable method for industrial nanocellulose production.

##### 3.1.3.2 Wet oxidation pretreatment

Wet oxidation pretreatment is a method that uses oxygen or air to decompose lignin and hemicellulose in lignocellulosic biomass under high pressure and alkaline conditions (typically with sodium hydroxide). High temperature and pressure increase the solubility of the oxidizing agent, accelerating dissolution [116,159]. The advantages of wet oxidation pretreatment include environmental benefits and minimal by-products, as it mainly involves water, oxygen, and a weak base, reducing reliance on toxic chemicals [166]. Saumita et al. [167] optimized wet oxidation parameters to enhance cellulose content and remove lignin. The optimal conditions were 185 °C, 0.5 MPa, and 15 min, resulting in a cellulose content of 66.97%, lignin removal rate of 89.18%, and hemicellulose solubility rate of 69.77%. However, the high cost of oxygen and catalysts limits the large-scale production of nanocellulose, a key factor for future research to address.

##### 3.1.3.3 Hot liquid water pretreatment

Hydrothermal water pretreatment does not require additional catalysts; the hydrated hydrogen ions act as weak acids to hydrolyze hemicellulose. This method treats biomass with high-pressure water at temperatures of 160-240 °C to decompose its structure [116,162]. Hydrothermal water pretreatment causes minimal environmental pollution and retains a high cellulose content. It also improves subsequent enzymatic hydrolysis and glucose recovery [168]. However, hydrothermal water is not effective in dissolving lignin, and additional methods are typically required for biomass with high lignin content. Moreover, this method requires specialized equipment for high-temperature and high-pressure operations, resulting in significant energy consumption. Additionally, water recovery and treatment need further consideration.

#### 3.1.4 Biological pretreatment

Biological pretreatment of lignocellulose is considered an environmentally friendly alternative to physical and chemical methods. This process utilizes microorganisms, such as bacteria, fungi, or enzymes, to decompose lignin and hemicellulose in biomass, relying on the natural biodegradation of the complex lignocellulosic structure [154,164]. White rot fungi, brown rot fungi, and certain bacteria selectively degrade lignin and hemicellulose. Additionally, microorganisms produce lignin-degrading enzymes, such as laccase and lignin peroxidase, to enhance lignin degradation [169]. Therefore, biological pretreatment is particularly attractive for applications with stringent requirements on residual chemicals, such as wound healing membranes [145] and drug delivery carriers [170]. However, its main limitations include slow reaction rates, sensitivity to processing conditions, and limited stability in large-scale implementation. As a result, biological pretreatment is generally more suitable for high-value biomedical materials rather than low-cost, large-volume production.

Overall, pretreatment of nanocellulose is not merely a matter of extraction efficiency; it requires a comprehensive balance among structural control, scalability, cost, and environmental impact. For example, chemically driven pretreatments that yield highly crystalline CNCs are advantageous as reinforcing phases in scaffold membranes or as drug carriers, but may raise concerns regarding reagent recovery and residual cytotoxicity [170]. Similarly, physicochemical pretreatment routes often involve substantial heat generation, posing environmental and safety challenges, while their complex processing requirements can limit economic feasibility in practical applications. In contrast, nanocellulose produced via mild enzymatic or oxidative pretreatment followed by mechanical disintegration is generally more suitable for porous scaffold membranes, as the resulting long fibrillar networks better support entanglement, water retention, and extracellular matrix (ECM)-like architectures [171]. Recent studies further indicate that selective pretreatment and controlled disassembly can produce highly flexible and tunable sub-nanofibrillar ribbons, which show promising potential in wound dressings, drug delivery, and bioactive scaffold systems [145,172].

### 3.2 Extraction process for nanocellulose

**Fig. 6:**
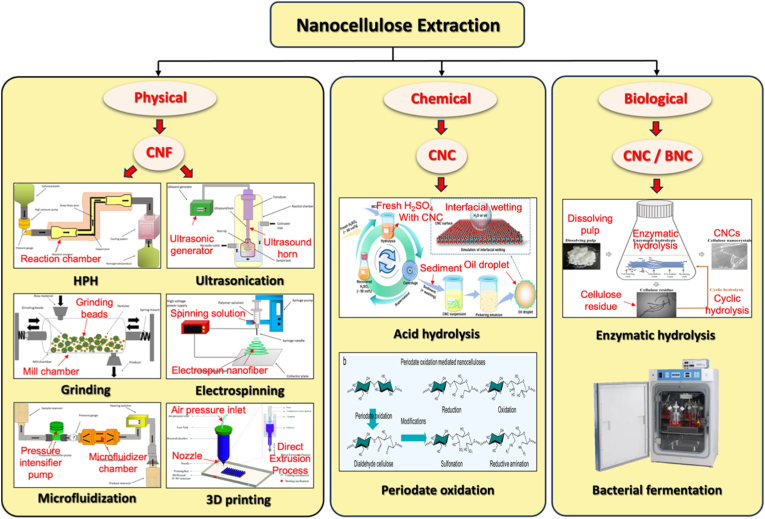
Summary of nanocellulose processing technologies.

The extraction of nanocellulose refers to the process of further breaking down, hydrolyzing and biosynthesizing purified or partially purified cellulose fibers to produce nanoscale building blocks. The extraction methods of nanocellulose usually involve physical, chemical, biological processes, or a combination of these techniques. Table 3 summarizes the extraction processes, products, and specific characteristics of various nanocelluloses. Different types of extraction methods are suitable for different types of nanocellulose, and Fig. 6 demonstrates the extraction technologies for different nanocelluloses.

**Table 3:** Summary of nanocellulose products.

Raw materials	Pre-treatment	Process	Products	Characteristic-	Properties	Ref.
Eucalyptus pulp	Oxalic acid treatment	High pressure homogenization	Oxalic acid based nanocellulose films (Ox-CNF films)	High specific surface area and ion exchange capacity Higher chemical stability	Fiber diameter: 6-20 nm; Length: 300-1200 nm; Limit of detection HgCl_2_: 20 μm	[173]
Piceaabies, *Pinus sylvestris*	Carboxymethylation	High pressure homogenization	Carboxymethylated microfibrillated cellulose films (MFC films)	Extremely low oxygen permeability; Excellent oil barrier properties; Increased oxygen transmission rate at high humidity	Fiber diameter: 5-10 nm; Crystallinity: 63 ± 8.6%; The air permeability: 0.2 nm/Pa·s; Optical transmittance: 90%	[95]
Pineapple peel waste	Bacterial fermentation	High pressure homogenization	BNC/graphite nanoplatelet composite membranes (BNC/GNP films)	High surface area but poor dispersion; High mechanical strength; Surface roughness optimization	Fiber diameter: 53.24 ± 27.05 nm; Tensile strength: 121.04 MPa; Elongation at break: 3.72%; The average roughness: 1.06 ± 0.15 μm	[44]
*Eucalyptus globulus*	TEMPO-mediated oxidation	High pressure homogenization	CNF/Chia mucilage matrix composite films	Enhanced mechanical properties; High hydrophobicity, antioxidant and antimicrobial properties	Fiber diameter: 26.3 ± 0.12 nm; Optical transmittance: 76-80%; Ultimate tensile strength: 25 MPa Water contact angle: 101°;	[174]
Eucalyptus	Enzymatic hydrolysis	Grinding process	CNF films	Mechanical properties decrease with increasing enzyme digestion time; High transmittance; High thermal stability	Fiber diameter: 9.7 ± 6 nm; Crystallinity: 58.0%; Optical transmittance: 71.09%; Tensile strength: 7.5-58 MPa; Young's modulus: 0.25-1.75 GPa	[26]
Hardwood bleach kraft pulp	DES esterification	Extrusion and colloid milling	CNF/Polylactic acid composite materials (CNF/PLA)	Improved bending and tensile strength; Improved dispersion and interfacial compatibility	Fiber diameter: <100 nm; Degree of polymerization: 300-500; Ultimate flexrual strength: 191 MPa; Ultimate tensile strength: 60 MPa	[164]
Spruce wood	Hydrothermal treatment and carboxymethylation	High-shear blending	Wood CNFs films	High degree of polymerization(DP); Excellent mechanical properties	Fiber length: 3.8 μm; DPv: 1026-1333; Tensile strength: 238 MPa; Toughness: 21.1 MJ/m^3^; Elongation at break: 14.6%	[175]
*Stipa tenacissima*	Sulfation in H_2_SO_4_/urea	Microfluidization	Sulfation nanocellulose films (S-NC films)	Environmentally friendly and efficient Excellent dispersion and transparency	Fiber diameter: 6.85 ± 3.11 nm; Tensile stress:85 ± 3.1 MPa; Elongation at break:7.36 ± 0.95%; Young's modulus:6.5 ± 0.292 GPa; Optical transmittance:80%	[176]
Pine pulp	DES esterification	Microfluidization	Hydrophobic cellulose nanofibers films (HCNFx fims)	Excellent hydrophobicity; Excellent tensile strength and plasticity; Recyclable and reusable	Fiber diameter: 6.5-8.2 nm; Tensile strength: 150.8 MPa; Young's modulus: 8.5 ± 0.5 GPa; Water contact angle: 153°	[177]
Spruce wood	Etherification	Microjet homogenization	CNF/lignosulfonic acid composite films (CNF/LA films)	Excellent mechanical properties and optical transparency; Excellent UV absorption, thermal oxidative ageing and water resistance	Fiber diameter/length: 20/950 nm; Tensile strength: 249 ± 6 MPa; Toughness: 23.6 ± 1.3 MJ/m^3^; Elongation at break: 14-17%; Young's modulus: 3.7-4.5 GPa	[178]
Poplar and Pine	Lignin Removal	Mechanical pressing	Anisotropic, transparent films with aligned CNFs	Excellent optical properties and mechanical strength; Anisotropic fluid transport	Fracture strength: 350 MPa; Toughness: 7.38 MJ/m^3^; Optical transmittance: 90%	[179]
Tunicates	Sulfuric acid hydrolysis or TEMPO-mediated oxidation	Ultrasonic processing	Tunicates CNF films	High crystallinity and mechanical stiffness; Highly anisotropic thermal conductivity	Fiber diameter/length: 15.5/1590 nm; Crystallinity index: 85.6-94.2; Ultimate Tensile strength: 205 MPa; Ultimate Toughness: 0.2 MJ/m^3^; Thermal conductivity: 2.37 W/mK	[47]
Cotton	TEMPO-mediated oxidation	Ultrasonic processing	Carboxymethyl cellulose/SiO_2_ nanoparticles/CNFs composite films (CMC/SiO_2_/CNFs films)	High mechanical strength; High thermal stability and flame retardant properties	Tensile strength: 120.98-151.62 MPa; Elongation at break: 10.57-12.96%; Young's modulus: 1.23-1.67 GPa; The peak of total heat release: 134 W/g	[180]
Bagasse and pine bark nuggets	Microwave-assisted deep eutectic solvents	Ultrasonic processing	LCNFs/polyanionic cellulose (PAC) composite films	Good suspension stability; Good mechanical properties; UV resistance; Environmentally friendly	Tensile strength: 55.8 ± 1.1 MPa; Tensile modulus: 646.7 ± 4.6 MPa Elongation at break: 26.3 ± 0.6%; UV transmittance: 2.9%;	[181]
Wood chips and cotton	Polymers blending	Vacuum filtration with ultrasonic processing	CNC/polyethylene glycol/graphene oxide composite films (CNC/PEG/GO films)	Excellent UV blocking properties; Good transparency; Improved mechanical properties; Preservation of chiral nematic structure	Fiber diameter: 11.2 ± 3 nm; Tensile strength: 37.59 ± 3.08 MPa; Tensile modulus: 13 GPa; Optical transmittance: 78.4%; UVA blocking: 98.3%	[34]
Peanut shells	Alkali hydrolysis and ultrasonic treatment	Electrospinning process	Nanocellulose- polyvinyl alcohol (PVA) composite films	High specific surface area Excellent dialysis and adsorption properties	Fiber diameter: 48.67 nm; Distance between needle and collector: 10-15 cm	[40]
Wheat straw	TEMPO-mediated oxidation	Electrospinning process	Electrospun cellulose nanofibers (ECNFs)	Excellent water absorption; Low ion sensitivity;	Fiber diameter: 79-98 nm; Maximum water absorption: 225 g/g	[182]
Paper pulp	Carboxymethylation combined with high pressure homogenization	Electrospinning process	Aligned nanocellulose films	Partially aligned CNF; High optical transparency; Anisotropic humidity response; Reliable mechanical properties	Optical transmittance: 93%; Tensile strength: 100-166 MPa; Strain at break: 1.1-2.9%; Young's modulus: 9.6-12.4 GPa;	[183]
Ramie	Purification and acid hydrolysis	Electrospinning process	Nanofiltration membranes of PVA nanofibers and CNC	Excellent mechanical properties; Good thermal stability	Fiber diameter: 2-15 nm; Tensile strength: 18.9-34.23 MPa; Elongation at break: 49.5-64%; Thermal degradation temperature: 300°C-340 °C	[184]
Rice husk	TEMPO-mediated oxidation	3D printing	CNF/Silica nanoparticles films	Increased transparency in the wet state; High dimensional stability; Print applicability	Optical transmittance: 92-95%; Water contact angle: 23-35°; Ultimate Tensile strength: 58 MPa;	[185]
Wheat straw	TEMPO-mediated oxidation	Alkali treatment with ultrasonic processing	Steam modified Boron Nitride Nanosheets/CNF composite films (SM-BNNS/CNF films)	High solar radiation and thermal conductivity; Excellent mechanical strength and hydrophobicity, optical transparency	Tensile strength: 43.5 MPa; Elongation at break: 5.3%; Thermal conductivity: 2.103 W/mK Solar reflectivity: 89.5%	[186]
Pineapple	Bleach and alkali treatment	Sulfuric acid hydrolysis	Pineapple peel nanocellulose films (PPNc films)	High crystallinity and thermal stability; High mechanical strength	Fiber diameter/length: 15/189 nm; Crystallinity: 61.19%; Tensile strength: 3.32 MPa; Elongation at break: 6.7%	[33]
Sargasso	Alkali treatment	Hydrochloric acid treatment	KH560 modified CNF/polyvinyl alcohol composite films (KCNF/PVA films)	Improved mechanical properties; Excellent thermal stability, light transmission and regeneration properties; Good water resistance	Fiber diameter: 4-6 nm; Optical transmittance: 75%; Dry tensile strength: 249 MPa; Wet tensile strength: 50 MPa; Elongation at break: 320%; Water contact angle: 119°	[41]
Licorice residues	Bleaching, mechanical refining and enzymatic pretreatment	TEMPO-mediated oxidation	Chitosan nanofibers/lignin nanoparticles-CNF composite films (CHN/LNPs-CNF films)	Highly crystalline, thermally stable and hydrophobic; Good mechanical properties and UV-blocking ability	Fiber diameter: 130 nm; Crystalline index: 73.17%; Tensile stress: 13-32.5 MPa; Tensile strain: 1.15-1.65%; Optical transmittance: 76.3%	[28]
*Camellia oleifera*	DMAc/LiCl system	TEMPO-mediated oxidation	Tea Saponin-Camellia nanocellulose films (TS-TOCN films)	Excellent mechanical, oxygen barrier, antimicrobial and antioxidant properties	Fiber diameter: 419.3 nm; Tensile strength: 34.4-63.9 MPa; Elongation at break:1.8-4.7%; Young's modulus:1.4-4.6 GPa; Oxygen permeability: 2.88 cc·m^−2^.d^−1^	[94]
Corn husks	Bleach and alkali treatment	TEMPO-mediated oxidation	Highly transparent hydrophobic nanocellulose films	High transparency; Excellent hydrophobicity; Good stain resistance; Improved thermal stability	Fiber diameter: 15-20 nm; Crystallinity: 64.5%; Optical transmittance: 84.4-87.8%; Water contact angle: >150°;	[39]
Miscanthus x. Giganteus	Bleach treatment	Ammonium persulfate and TEMPO oxidation	MxG-CNF-CO_2_H/PVAc composite films	Good thermal stability and dispersibility; Increased mechanical strength; Lower yield	Fiber diameter: 3.8 ± 0.8 nm; Tensile strength: 91.63 ± 4.07 MPa; Young's modulus: 2.41 ± 0.29 GPa; Elongation: 4.12 ± 0.97%	[32]
Cotton and flax	Sulfuric acid hydrolysis	Wet cross-linking process	CNC/Amino-aldehyde films	Good transparency and haziness; Good waterproof performance;	Optical transmittance: 72-83%; Crystallinity index: 83.5-93.6% Ultimate Tensile strength: 45 MPa; Ultimate young's modulus: 11 GPa	[187]
*Gluconacetobacter xylinus*	Pre-culture in seed agar	Static culture	BNC films	Good crystallinity and thermal stability; Cultivation style affects performance	Fiber diameter: 20-40 nm; Yield: 2.48-3.97 g/L; Tensile strength: 0.234-0.264 MPa; Elongation at break: 12.1-28.9%; Young's modulus: 0.92-1.968 MPa	[44]
*Gluconacetobacter xylinus* NCIM 2526	Pre-vaccination	Stirred culture	BNC films	High yield; High sugar conversion ratio; High purity and hydrophilicity	Fiber diameter: 66 ± 20 nm; Yield: 6.6 ± 0.3 g/L; Sugar consumption rate: 90.0 ± 3.3%;	[188]
Bagasse	TEMPO-mediated oxidation	3D printing	CNF-Alginate 3D printing bio-ink	Non-cytotoxic; Good cross-linking with Ca^2+^; Stable 3D printed structures	Carboxylic acid content: 1128 ± 36 μmol/g; Cell viability: 70-80%; Conductivity: 400 μs/cm	[189]
Wood pulp	High speed mixing	3D printing	CNF/Alg/CaCO3 nanocomposite ink	Good structural stability; Highly adjustable mechanical properties; Good biocompatibility	Fiber diameter: 20 nm; Tensile stress: 4.2 MPa; Tensile modulus: 23.4 ± 1.1 MPa;	[190]
Waste wood scraps	Dewaxing, bleaching	Homogenization with ultrasonic processing	Urea-loaded CNF/carboxyl methyl cellulose hydrogel (UCNF)	Good water absorption and water retention; Good biodegradability; Release slow fertiliser	Water absorption capacity: 147 g/g; Urea loading capacity: 92%; Weight loss in bacterial environment for 3 months: 81%	[191]
Energy cane Bagasse	Microwave assisted natural deep eutectic solvents	Microfluidization	LCNFs gel	Green and sustainable (DES recyclable); High crystallinity; Excellent rheological properties and durability; Improved magnetorheological properties	Fiber diameter: 8.49 nm; Crystallinity: 55.8%; Viscosities: 1436.1 mPa s(0.1T); NADES recovery ratio: 93.5%	[192]
Soy hull	Acid hydrolysis and ultrasonic treatment	Freeze-thaw cycle	SHNC/PVA/SA/Tannic acid (TA) composite hydrogel	Excellent mechanical properties; High water retention properties; Excellent antimicrobial properties	Compressive strength: 10 kPa; Tensile strength: 0.35 MPa; Elongation at break: 160%; Water contact angle: 30.06°; Cell viability: >75%	[193]
Wood pulp/cotton	Acid hydrolysis	Sol-gel process	CNC/Fe_3_O_4_/Co_3_O_4_ aerogel	Ultraparamagnetic; High ratio capacitance; Excellent cycling stability	Compressive modulus: 3240 kPa; Maximum magnetization value: 17.8 ± 0.1 emu/g; Maximum specific capacitance: 294 F/g	[194]
Birch Kraft Pulp	Grinding	Freeze drying	Phthalimide-modified CNF aerogel	Increased surface area; Increased CO_2_ adsorption capacity	Fiber diameter: 20-50 nm; BET surface area: 335 m^2^/g; CO_2_ adsorption: 5.2 mmol/g;	[195]
Milk-container board	DES pretreatment	Freeze drying	MCB-CNF aerosol	Enhanced hydrophobicity; Highly efficient filtration performance	Porosity: 99.1%-99.8%; BET surface area: 5.9-18.6 m^2^/g; Quality factor: 2.1 × 10^−7^ m	[196]
Tunicates	Bleach and alkali treatment	Sulfuric acid hydrolysis	CNC/Rubber composite aerogel	High mechanical stability; Excellent solvent resistance and self-cleaning properties; Excellent thermal insulation	Fiber diameter: 16.6 nm Porosity: 98.2-99.3% BET surface area: 31.0-136.5 m^2^/g; Water contact angle: 117.7-131.0°;	[48]

(Adapt from Refs. [120,197] with permission. Copy right Taylor & Francis, 2024)

#### 3.2.1 Physical method

Several physical techniques, including high-pressure homogenization, ball milling, microfluidization, high-intensity ultrasound, electrospinning, and 3D printing, are commonly used for extracting CNF. High-pressure homogenization, ball milling, and microfluidization are traditional methods that apply various mechanical forces to break down cellulose fibers into nanoscale sizes and are widely used in industrial applications. CNF membranes produced by these methods typically exhibit uniform fiber size, excellent mechanical properties, and high transparency [157,166,198]. Although mechanical processing is scalable and cost-effective, it requires substantial energy input and can cause fiber degradation if not carefully controlled [199]. It has been reported that in 2023, the average electricity price in the United States was 0.168 USD/kWh, and the production cost of mechanically extracted nanocellulose is strongly affected by electricity prices. The energy consumption of conventional HPH (high-pressure homogenization) pretreatment can reach 30–50 kWh/kg [188,189]. For nanocellulose products intended for biomedical applications, it is also essential to ensure clean physical processing without residual functionalization, which further restricts the scalability of producing nanocellulose solely through physical methods. To address this, high-intensity ultrasound is often used to assist in nanocellulose extraction, improving CNF size uniformity, decomposition efficiency, and yield [46,170,171].

Electrospinning and 3D printing are emerging technologies for nanocellulose extraction, gaining increasing attention from researchers. Electrospinning is a method for preparing nanofibers by jetting a polymer solution or melt through a high-voltage electric field. In the high-voltage electric field, the cellulose solution is sprayed from the spinneret. As the solution travels, the solvent evaporates, and cellulose filaments are deposited on the collector to form a non-woven fiber mat [190,191]. The resulting nanocellulose exhibits good mechanical properties and biocompatibility, making it suitable for applications in tissue engineering, drug delivery, protective clothing, and wound dressings [192,193]. Several methods for preparing nanocellulose using 3D printing are available, including direct ink writing (DIW), matrix-assisted printing, and stereolithography [200]. Researchers have mixed various substances into nanocellulose-based hydrogel bioinks for 3D printing complex geometric structures [201]. Nanocellulose often serves as a reinforcing material, combining with substances like polylactic acid to form composite filaments, which significantly enhance the thermal stability and mechanical properties of the composites [195,196]. Furthermore, there is increasing research into more efficient, simpler, and environmentally friendly 3D printing methods for preparing nanocomposites. These methods allow for personalized customization of raw materials and the spatial structure of the final product, opening new research directions in metal-organic frameworks and cell culture scaffolds [199,202].

#### 3.2.2 Chemical method

Unlike physical methods that rely on shear force to break down fibers, chemical methods selectively break the glycosidic bonds in amorphous cellulose. Acid hydrolysis is a widely used chemical method for extracting CNCs. It typically uses strong inorganic acids, such as sulfuric acid and hydrochloric acid, to hydrolyze the non-crystalline regions of cellulose, preserving the crystalline regions to produce CNCs with high crystallinity [203]. CNCs prepared by this method exhibit high mechanical strength, making them suitable for applications such as reinforcing materials [32,39]. However, traditional acid hydrolysis has several drawbacks, including severe equipment corrosion, environmental pollution, and high-water consumption. To address these issues, researchers have explored more sustainable acid hydrolysis methods. For example, solid acid hydrolysis, an emerging technology, uses organic solid acids (e.g., citric acid, oxalic acid) or inorganic solid acids (e.g., phosphotungstic acid, phosphoric acid) for hydrolysis. These methods are less corrosive, require milder conditions, and allow for acid recovery and reuse, thereby reducing environmental pollution and production costs [204]. Overall, acid hydrolysis remains crucial in nanocellulose preparation. However, with increasing emphasis on environmental protection and sustainability, more eco-friendly and efficient acid hydrolysis technologies are being developed and optimized.

Beyond the ongoing efforts to optimize acid hydrolysis techniques, increasing attention has been directed toward developing alternative chemical oxidation methods to replace TEMPO-mediated oxidation for the extraction of nanocellulose. Among these, periodate oxidation has emerged as a promising approach, particularly due to its potential applications in the field of biocatalysis. In aqueous environments, sodium periodate selectively cleaves the C2–C3 bond of the anhydro glucose unit (AGU) in cellulose, generating two highly reactive aldehyde functional groups on the cellulose surface [197]. These dialdehyde groups significantly expand the potential for post-modification of nanocellulose, offering a versatile platform for functionalization.

Adam Rees et al. [205] successfully developed a bio-ink based on periodate-oxidized nanocellulose and fabricated a 3D-printed scaffold membrane suitable for wound dressing applications. Compared to TEMPO-oxidized nanocellulose, the periodate-oxidized counterpart exhibits higher consistency and favorable rheological properties. Its internal three-dimensional structure presents a more open porous network, contributing to enhanced antibacterial performance. Despite these advantages, periodate oxidation significantly affects the crystallinity of cellulose, and alterations in the polymorphism and morphology of the resulting cellulose crystals remain topics that warrant further investigation [206].

#### 3.2.3 Biological method

Enzymatic hydrolysis is a biologically assisted processing method that utilizes the specific catalytic activity of cellulase enzymes to hydrolyze the amorphous regions of cellulose while preserving the crystalline domains. It is important to note that enzyme-assisted hydrolysis for nanocellulose extraction, as discussed in this section, is conceptually distinct from enzymatic pretreatment. In pretreatment, enzymes are primarily used to enhance the accessibility of cellulose, whereas during extraction they directly assist in or regulate the nanoscale fibrillation process. Although these roles are related, they are not equivalent. This extraction method offers advantages such as mild reaction conditions, environmental friendliness, and the ability to produce nanocellulose with an intact structure [207]. Studies have shown that enzymatic treatment can achieve a nanofibril yield as high as 69%, which is substantially higher than that obtained from purely physical methods (21%). Moreover, the enzymatic microfluidization process generates only 4.3 kg CO_2_-eq/kg CNF, indicating a relatively low overall environmental burden [188,189].

However, enzymatic hydrolysis is generally less efficient, requiring longer reaction times and higher costs. The low yield of nanocellulose produced by isolated enzyme hydrolysis is also one of the limitations of current research. Researchers have employed collaborative physical methods, the addition of catalysts, and the optimization of customized enzyme combinations to enhance the feasibility of enzyme hydrolysis of nanocellulose for medical and energy applications. Zhao et al. [208] regulated the structure of nanocellulose using endoglucanase and xylanase at varying loadings, and enhanced processing efficiency through ball milling-assisted treatment. This approach yielded up to 24.4% xylo-oligosaccharides and 21.5% nanocellulose containing cellulose II-type allomorphs. Li et al. [209] employed an uncharacterized thermophilic My lytic polysaccharide monooxygenase (MtLPMO9V) in synergy with cellulase to achieve efficient hydrolysis of lignocellulose. Compared to the wild-type MtLPMO9V, the engineered enzyme exhibited an 89% increase in catalytic efficiency and a 55% improvement in the saccharification efficiency of cellulose substrates. Future efforts are expected to focus on optimizing enzymatic treatment strategies to produce functionalized nanocellulose, aiming at applications in biomedical carriers and smart materials [210].

Bacterial fermentation is a biosynthetic method for producing BNC, with two main production processes: static culture and agitated culture [101]. In static culture conditions, bacterial cultures grow in shallow, fixed containers with a nutrient medium, where BNC forms a uniform, highly organized multilayer membrane-like structure. Although the production rate is slow, the method is simple, the structure is well-ordered, and it exhibits excellent mechanical properties [45]. In agitated culture conditions, bacterial cultures grow in a bioreactor or shake flask with continuous or intermittent stirring. BNC tends to form small clumps and fragments, which increases yield but reduces some physical properties. This form is suitable for use as bulk material or thickeners [188]. In summary, the choice of biological method for BNC production depends on the desired characteristics and intended applications. Static culture is ideal for producing structured films for biomedical applications, while agitated culture is better suited for industrial-scale production focused on high yield.

## 4 Surface modification methods for nanocellulose membranes

Source selection and fabrication technology provide the structural and chemical basis for later surface modification, while surface modification further tailors membrane performance toward specific biomedical requirements. In practical applications, enhancing the surface properties of nanocellulose is crucial for expanding its functionality and range of applications. Table 4 lists various surface modification methods for nanocellulose and their specific applications. These methods can be categorized into two main types: chemical and physical. Chemical modification involves replacing hydroxyl (OH) groups on the nanocellulose surface with functional groups via processes [238]. In contrast, physical modification techniques include surface adsorption, ionic liquid welding and plasma treatment [239]. There are also some biochemical modification techniques, including enzymatic reactions and biological extracts.

**Table 4:** Summary of nanocellulose modification and applications.

Modification method	Type	Chemical sources	Modified Characteristics	Applications	Ref.
Grafting	CNC	Glycerol monomethacrylate, dimethyl aminoethyl methacrylate, butyl methacrylate	Rapid and efficient self-healing properties; Young's modulus, elongation at break, tensile strength and toughness increased significantly; Optimized thermo-mechanical properties	Self-healing nanocomposites	[211]
		Poly(acryloyl hydrazide)	Highly efficient Cr(VI) adsorption capacity; Optimized adsorption conditions; Excellent reusability	High-performance adsorbents for the removal of harmful underwater pollutants	[212]
	CNF	0.4 wt% 25 mL Tea Saponin 0.1g DMAP (catalysts)	Improved tensile strength; Significantly improved antimicrobial properties; Reduced oxygen transmission rate	Food and pharmaceutical field	[94]
		N-(3-(Dimethylamino)propyl)-N′-ethylcarbodiimide hydrochloride, N-hydroxysuccinimide	Potential for biofunctionalization; Enhanced biological activity	Biomedical devices, wearable sensors and drug-releasing materials.	[213]
Esterification	CNF	Triethylmethylammonium chloride, imidazole, Dodecenylsuccinic anhydride	Excellent hydrophobicity; Renewable Recycling; Easy conversion of 3D shapes in the wet state	Novel environmentally friendly water-plasticised biomaterials	[177]
		Choline chloride, formic acid, lactic acid, acetic acid, malonic acid, oxalic acid dihydrate, citric acid	Higher yield; Improved mechanical strength; Improved dispersion and interfacial compatibility	Polymer Composite Reinforcements	[164]
Silanization	CNF	Methyltrimethoxysilane (MTMS)	Wettability from hydrophilic to hydrophobic; Photosynthesis applicability; Improved mechanical properties	Agricultural thermal management	[186]
		γ-propytrimethoxysilane (KH560)	Increased tensile strength (3.49 → 6.65 MPa); Significantly reduced water vapor permeability and improved water resistance	Green and renewable packaging materials	[214]
		3-mercapto-propyl-trimethoxysilane (KH590)	Improved interfacial bonding of CNF to PVA; Increased hydrophobicity; Increased CNF dispersion	Recycled composites in humid and aqueous environments	[40]
		Methyltrimethoxysilane, hexadecyltrimethoxysilane	Increased porosity; Improved filtration efficiency; Enhanced hydrophobicity	High Efficiency Particulate Filter	[196]
		3-mercapto-propyl-trimethoxysilane (KH590)	Improved dispersion; Significantly improved interfacial bonding	Cementitious composite materials	[215]
Sulfonation	CNC	1,4-butane sulfonic acid ester	Improved dispersion and compatibility; Increased tensile strength (41.53%) and elongation at break (22.18%); Enhanced antimicrobial properties	Degradable PLA composites	[216]
	CNF	Sulfosuccinic acid (SSA)	Increased water absorption capacity; Reduced methanol permeability; Increased Young's modulus but decreased strength and elongation at break; Membrane products are more brittle and hard	Solid Electrolyte Membrane for Direct Methanol Fuel Cell (DMFC)	[217]
		Bisulfite	Better anti-coagulant properties; Low haemolytic activity	Complementary system in blood serum	[218]
TEMPO-mediated oxidation	CNF	TEMPO, Laccase, O_2_	Improved wet tensile strength; Increased carboxyl group content	High performance films and hydrogels	[219]
		TEMPO, NaBr, NaClO	Increased surface charge density; Increased permeability; Increased retention of high-valent ions	Low-energy water purification materials	[220]
Phosphorylation	CNF	Diammonium hydrogen phosphate	Improved cycling and multiplication performance of Li-S batteries; Inhibited polysulfide shuttle effect	High-performance Li-S batteries	[221]
Carbamation	CNF	Phthalimide	Increased specific surface area; Reduced porosity; Improved CO_2_ adsorption performance; Reduced thermal stability	Air filtration	[195]
Carboxymethylation	CNC	Sodium ethanol, monochloroacetic acid	Morphology changed from short rods to spherical particles; Increased dispersion stability; Optimization of surface modification effects	Composite Materials, packaging	[144]
Surfactant	CNC	Lauroylarginine (LAE)	Increased surface hydrophobicity; Better dispersion and interfacial compatibility; Reduced aggregation and interfacial debonding of nanoparticles	Nanofiller materials	[222]
		Cetyltrimethyl ammonium bromide (CTMAB)	Better dispersion and interfacial compatibility; Improved mechanical properties (Tensile strength, elongation at break and tear strength increased by 132.8%, 20% and 66.1% respectively); Improved accelerated vulcanisation and ageing resistance	Polymer matrix composites with nanocellulose as reinforcing filler	[38]
		Sodium alkyl naphthalene sulfonate (Surfom WG 8168)	Increased stability and dispersion; High electrostatic stability; Slightly better thermal stability	Coatings, emulsifiers and nanocomposite reinforcements	[223]
		Cetyltetramethylammoniumbromide (CTAB); Dimethyldidodecylammonium bromide (DDAB); Dimethyldihexadecylammonium (DHAB)	Improved dispersion; Higher hydrophobicity; Excellent corrosion protection properties	Nanocomposites for corrosion resistance	[224]
	CNF	DTAB, SDS, SLES	Increased modulus of elasticity at low concentrations; Significant effect on rheological properties	Neat CNF suspension	[225]
		Polybutylene succinate	Increased tensile strength(24 → 28 MPa); Increased elongation(4.5%→6.1%); Increased toughness(0.625 → 1.05 kg/mm^2^); Improved dispersion	Sustainable and environmentally friendly nanofillers	[226]
Wet cross-linking process	CNC	Dimethylol-dihydroxy-ethylene-urea	Improved water resistance; Improved tensile strength and modulus(45 MPa and 11 GPa); Increased transparency	Waterproof material	[177]
Ionic liquid modification	CNC	N-methylmorpholine-N-oxide (NMMO)	Faster response to humidity changes; Extended range of response colours;	Sensing, anti-counterfeiting and decorative fields	[227]
		1-hexadecyl-3-methylimidazolium bromide [C_16_mim]Br	Enhanced dispersion stability; Improved lubrication properties	Bio-based lubricants	[228]
		[BMIm][BF_4_]	Improved thermal stability; Improved redispersibility; Improved mechanical properties	Toughening filler for PLA	[229]
		[VBIm][BF_4_]	Chiral liquid crystal structure; Stable dispersion	Reinforced nanocomposites	[230]
	CNF	[DBNH][OAc], gamma-valerolactone	Smooth, non-agglomerated surface characteristics; Faster degradation; Reduced water absorption; Enhanced mechanical properties	Bioplastics with accelerated degradability	[231]
Tunable wet-drawing alignment and ionic cross-linking method	CNF	Soft waterborne polyurethanes	Significant increase in tensile strength (323 → 715 MPa) and toughness (110 → 186 MJ m^−3^); More structured and tightly packed fibers	Functional textiles and military aerospace materials	[232]
Enzymatic modification	CNC	Lipase, [BMIm]HSO_4_, [BMIm]BF_4_, methyl laurate	Increased yield; Increased crystallinity; Improved thermal stability	Biodegradable green materials	[233]
	CNF	a-Chymotrypsin, glutaraldehyde, ethyl(dimethylaminopropyl) carbodiimide, ammonium sulfate, amine-functionalized magnetic nanoparticles	Improved enzyme loading and stability; Enhanced enzyme activity; Immobilized enzymes can be recovered and reused	Immobilized enzyme applications	[234]
Bio-extracts modification	BNC	*T. arjuna*, *A. indica*, *W. somnifera*, *T. cordifolia*, *M. koenigii*	Better antimicrobial properties; Higher moisture content, water retention value and high porosity	Antimicrobial wound dressings, herbal masks, tissue-engineered scaffolds, drug delivery	[235]
	BNC	Pomegranate peel extract, green tea extract, rosemary extract	Better antimicrobial and antioxidant properties	Food shelf life extension	[236]
Aldehyde functionalization	BNC	Sodium periodate (NaIO_4_), Ethylene glycol	Enhanced compression modulus (35 ± 3 → 98 ± 7 kPa); Self-healing ability; High thermal stability;	3D printed tissue engineering scaffolds	[237]

**Fig. 7:**
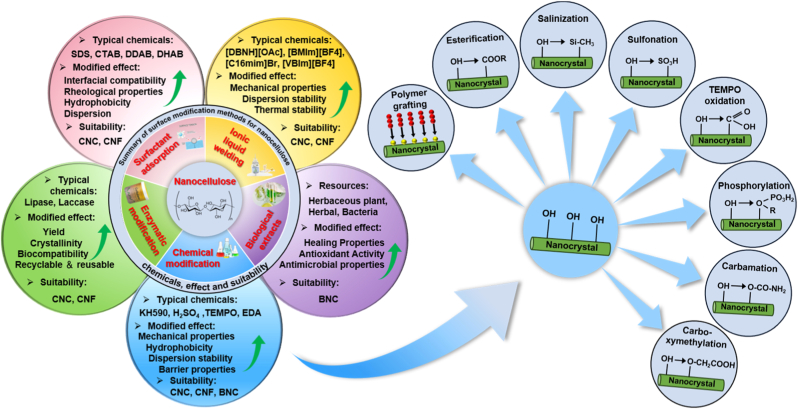
Summary of surface modification methods for nanocellulose.

The choice of surface modification method depends on the type of nanocellulose and the desired application. For biomedical scaffold membranes, surface modification of nanocellulose not only alters its dispersibility and interfacial compatibility, but also directly regulates the biological microenvironment at the material surface. Variations in surface functional groups, charge density, wettability, and roughness can significantly influence cell adhesion, tissue regeneration, protein adsorption, and antibacterial performance. Therefore, when evaluating the biomedical suitability of surface-modified nanocellulose membranes, it is essential to consider reaction selectivity, removal of residual reagents, and the resulting surface chemistry. Fig. 7 summarizes the surface modification approaches for various types of nanocellulose.

### 4.1 Chemical modification

Chemical modification of nanocellulose involves introducing new functional groups through chemical reactions, which alters its surface properties and chemical composition. Hydrophilicity, dispersibility, and interfacial compatibility are factors that hinder nanocellulose's application in certain industries [240]. Chemical modification reduces the influence of intramolecular and intermolecular hydrogen bonds in nanocellulose, promoting a uniform distribution of nanoparticles. This method significantly improves its mechanical strength and interfacial functionalization [203].

#### 4.1.1 Grafting

**Fig. 8:**
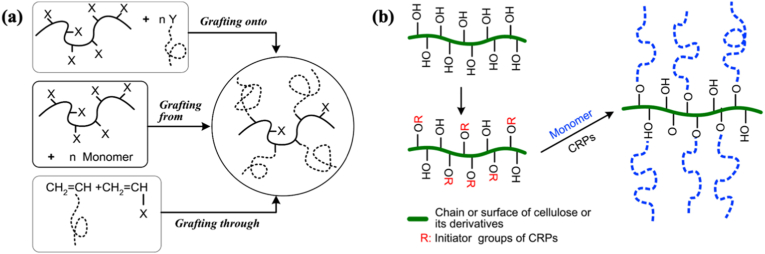
Schematic diagram of the mechanism of graft copolymer synthesis (a) methods for synthesis of graft copolymers (b) Grafting from approach for the graft modification of cellulose.

Graft polymerization using free radicals is one of the most widely used methods for chemically modifying nanocellulose by introducing polymer chains onto its surface. Common monomers include acrylates, styrene, and methacrylates [203]. Other grafting techniques include atom transfer radical polymerization (ATRP), reversible addition-fragmentation chain transfer (RAFT), nitrogen oxide-mediated free radical polymerization (NMP), and ring-opening polymerization (ROP) [241]. Fig. 8 illustrates the schematic mechanism of graft copolymerization onto nanocellulose. From a mechanistic perspective, grafting modifies the biological characteristics of the nanocellulose surface by replacing the hydroxyl-dominated interface with a polymer-functionalized interface. This transformation enables the regulation of steric stabilization, hydration layer formation, local charge distribution, and the presentation of bioactive moieties, thereby influencing protein adsorption and subsequent cellular responses. Anam et al. [211] prepared rapidly self-repairing CNC composites at low temperatures via simple graft copolymerization. The composites exhibited a tensile strength of 25.49 MPa and a toughness of 2539 MJ/m^3^. Sang-Hee Park et al. [212] grafted polyacrylamide (PAH) chains onto the CNC surface using ATRP, achieving efficient Cr(VI) adsorption with a maximum capacity of 457.6 mg g^−1^. Different monomer polymers and grafting processes also have varying effects on nanocellulose, contributing to the creation of advanced materials with specialized functions.

(Adapt from Ref. [242])

However, the graft polymerization process can introduce new chemical groups and potential impurities, which may alter the material's biocompatibility and cytotoxicity. Strategies such as selecting biologically derived grafts and controlling grafting density can help minimize adverse cellular effects. Mariia Stepanova et al. [243] prepared composite films using poly(glutamic acid) (PGlu)-functionalized CNCs and PLA as the matrix polymer. After subcutaneous implantation in the dorsal region of rats for four weeks, the films demonstrated excellent biocompatibility, showing a 35–45% reduction in fibrous capsule thickness compared to pure PLA, and a two-fold decrease in blood vessel formation relative to unfilled materials, indicating the lowest inflammatory response. Olga Solomakha et al. [244] covalently modified CNCs with amphiphilic polyanions to improve their dispersibility in a polycaprolactone (PCL) matrix and enhance the osteoconductivity of the resulting composite films. The presence of lysine residues in PGlu contributed to a cell adhesion rate reaching 80% of the regeneration level. These findings indicate that grafting not only enhances compatibility with polymer matrices, but also modulates the adsorbed protein layer and subsequent cellular recognition. This strategy can therefore be further exploited in scaffold membranes for bone regeneration, wound healing, and implant–tissue interfaces [245].

#### 4.1.2 Etherification

Esterification is a process where the abundant hydroxyl groups on nanocellulose chains react with carboxylic acids or anhydrides to form ester groups, altering its surface properties. Controlling the degree of substitution and optimizing reaction conditions can enhance the application performance of nanocellulose in composite materials [203]. Recently, researchers have developed a novel method for esterifying and enhancing the hydrophobicity of CNF [177], utilizing the efficient dissolving ability and favorable reaction conditions of the DES system. The prepared CNF films have a maximum tensile strength of 150.8 MPa and exhibit good hydrophobicity, enabling dual recovery of films and reagents. This offers a new approach for developing environmentally friendly water-soluble bioplastics. Liu et al. [164] also employed the DES system to esterify and modify CNFs. The esterification reaction increased the surface hydrophobicity of CNFs, improving their dispersibility in non-polar solvents. Additionally, the introduction of ester bonds enhanced the interaction between CNFs and the PLA matrix. Experimental results show that the optimal bending strength of PLA composites containing 10% esterified CNFs reaches 191 MPa.

#### 4.1.3 Silanization

**Fig. 9:**
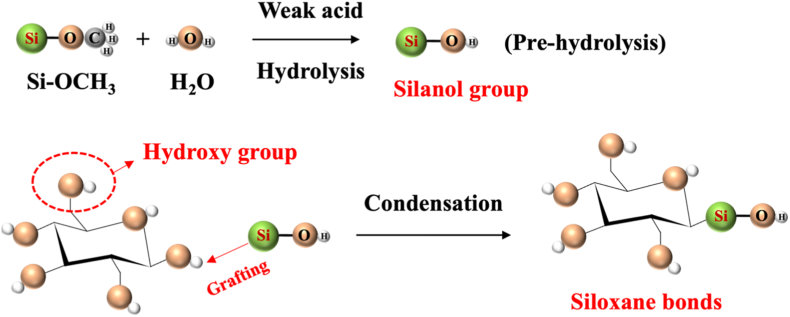
Schematic diagram of the mechanism of the silanization reaction process.

Silanization process involves the reaction of hydroxyl groups on nanocellulose with a silane reagent, introducing silicon-based functional groups (Si-OH) onto the surface to form stable siloxane bonds (Si-O-C) [246,247]. Fig. 9 shows a schematic diagram of the mechanism of the silanization reaction process. Common silane reagents include (3-aminopropyl) triethoxysilane (APTES), methyltrimethoxysilane (MTMS), and 3-mercaptopropyl-trimethoxysilane (KH590). Silanized nanocellulose typically exhibits strong hydrophobicity, and the resulting nanocomposites show significant improvements in thermal stability and flame retardancy [238]. Chen et al. [186] modified CNFs with MTMS vapor, producing CNF composite films with a tensile strength of 32.7 MPa, 2.04 times higher than that of the unmodified product. Their excellent mechanical properties and thermal dissipation performance make them suitable for agricultural heat management. Qin et al. [214] used the silane coupling agent KH560 as a reaction platform, bridging CNF and nano-silica particles (NS) to form a nanohybrid skeleton and introduce epoxy functional groups to enhance the interface interaction between the nanofillers and the soy protein isolate (SPI) matrix. Additionally, KH560 promoted the uniform dispersion of CNF and NS, reducing agglomeration and improving the mechanical properties and water resistance of the composite films.

For biomedical applications, precise control over the type of silane and the reaction conditions is essential. AMSS Nascimento et al. [248] investigated the effects of varying silanization levels of BNC on drug delivery performance. They found that an increase in pH enhanced the maximum ibuprofen adsorption capacity of silane-modified BNC to 98.92 mg/g. Notably, BNC modified with 70% APTES and formed into coatings under ambient drying conditions exhibited more stable drug responses and demonstrated excellent antibacterial activity. This suggests that silanization may be beneficial for drug delivery membranes and infection-control interfaces; however, its optimization requires a careful balance among drug loading capacity, antibacterial functionality, and acceptable cytocompatibility. In scaffold membranes, moderate silanization can enhance protein anchoring and interfacial stability, whereas excessive silanization may reduce water uptake and compromise the hydrated interface required for cell spreading and tissue integration.

#### 4.1.4 Sulfonation

Sulfonation is a chemical modification method that introduces a sulfonic acid group (-SO3H) onto the surface of nanocellulose, primarily for the production and modification of CNCs. Sulfonation reagents include concentrated sulfuric acid and sodium bisulfite [249]. Sulfonation enhances the ionic exchange capacity and hydrophilicity of nanocellulose, making it suitable for advanced applications, such as fuel cells and biological coagulation. Moreover, highly anionic surfaces can influence protein adsorption, coagulation pathways, and complement activation. The introduction of sulfonic groups is therefore particularly relevant for the design of blood-contacting or wound-related biomedical membranes. Arisara and Wunpen [217] prepared nanocellulose membranes modified with sulfonated succinic acid (SSA), resulting in a higher ionic exchange capacity and improved proton conductivity and methanol barrier properties. However, the mechanical properties of the product decreased after modification with a high concentration of sulfonic acid groups. Igor et al. [218] used sodium bisulfite to modify nanocellulose beads and compared the properties before and after modification. They found that sulfonated nanocellulose beads exhibited excellent anticoagulant properties. Although they did not effectively control complement system activation, they maintained good blood compatibility.

#### 4.1.5 TEMPO oxidation

**Fig. 10:**
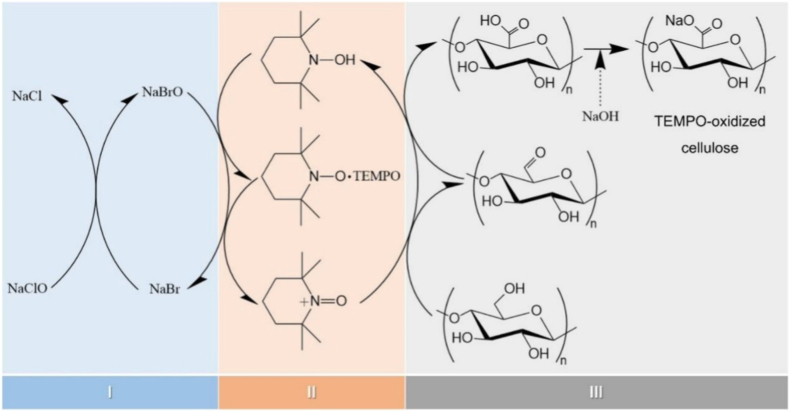
Reaction mechanism of oxidized nanocellulose in the TEMPO system.

**Fig. 11:**
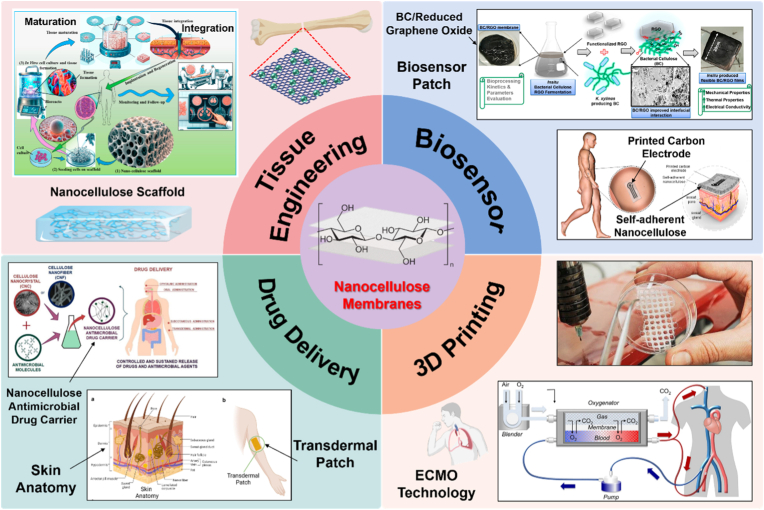
Biomedical applications of nanocellulose membranes.

Oxidation mediated by TEMPO is a simple and environmentally friendly method for chemically modifying nanocellulose. It selectively oxidizes the surface primary hydroxyl groups (C6-OH) to carboxyl groups (-COOH) [250]. TEMPO oxidation introduces a high content of carboxyl groups to nanocellulose, which ionize in water, resulting in a higher surface charge and improved dispersibility in aqueous media (Fig. 10). Carboxylated surfaces can improve wettability and promote serum protein adsorption in conformations favorable for cell adhesion. However, excessive oxidation may lead to over-swelling, local softening, and structural instability. Therefore, the degree of oxidation must be carefully controlled to avoid compromising membrane integrity. Bastien Michel et al. [252] prepared modified TEMPO-oxidized nanocellulose films and cold gels. The introduction of carboxyl groups enhanced the swelling capacity of the products, promoting drug diffusion and retention within the matrix. Additionally, the dissociation of carboxyl groups enhanced the modification effect, enabling drug release to be regulated according to pH changes. This makes modified TEMPO-oxidized nanocellulose a promising advanced material for sustained delivery of active ingredients (Fig. 11).

(Adapt from Ref. [251])

**(Adapt from**^172^,[334], [335], [336], [337], [338]**)**

Chen et al. [219] used the TEMPO/Laccase/O_2_ (TLO) oxidation system to modify nanocellulose from various plant sources. The carboxyl content in the modified materials ranged from 0.558 to 0.615 mmol/g, significantly improving the wet strength and mechanical properties of the resulting films and hydrogels. Joona et al. [220] applied TEMPO-mediated oxidation to modify cellulose ultrafiltration membranes, enhancing ion rejection and permeability. They also investigated the effect of different oxidant dosages on the degree of modification. FTIR and XRD analyses showed that the oxidation treatment had a minimal effect on the bulk chemical composition of the membrane, primarily affecting the surface. High exposure oxidation causes dissolution of the membrane surface material, resulting in increased permeability and molecular weight cutoff values. This provides new opportunities for low-energy, high-efficiency water purification technologies.

#### 4.1.6 Phosphorylation

Phosphorylation improves the physical and chemical properties of nanocellulose by introducing a high density of negatively charged phosphate groups (-PO_4_/-PO_3_) on its surface. Phosphate groups, due to their polybasic nature, exhibit stronger charge repulsion than carboxylate and sulfonate groups. They are commonly used in flame retardant and battery materials [253]. Li et al. [221] created an intelligent polysulfide barrier by phosphorylating nanocellulose, enhancing the durability of lithium-sulfur (Li-S) batteries. This functional film effectively inhibits the polysulfide shuttle effect and enhances lithium-ion transport, demonstrating excellent cycle stability and rate performance even under high sulfur loading and limited electrolyte conditions.

#### 4.1.7 Carbamation

Carbamation introduces a carbamate (urea-derived) group onto the surface of nanocellulose, typically by reacting it with urea or isocyanate compounds [227,247]. Nanocellulose modified by carbamation exhibits good stability and adsorption properties. Sima Sepahvand et al. [195] modified CNF with phthalimide to improve its CO_2_ adsorption capacity. The modified CNF aerogel achieved a maximum CO_2_ adsorption capacity of 5.2 mmol/g with 1.5% phthalimide content, offering a new approach for efficient, low-cost CO_2_ capture. However, studies show that current elemental analysis methods overestimate the free isocyanate content on the surface of nanocellulose, and that amination temperature negatively affects the number of free isocyanates. A lower temperature (e.g., 55 °C) is recommended to improve amination reaction efficiency [254].

#### 4.1.8 Carboxymethylation

Carboxymethyl-modified nanocellulose is produced by introducing carboxymethyl groups (-CH_2_COOH) onto the cellulose backbone, resulting in carboxymethyl cellulose (CMC). The reactant ratio, reagent amount, reaction temperature, and time can influence the carboxymethylation modification efficiency [255]. Nanocellulose modified by carboxymethylation generally exhibits good dispersibility and viscosity. The modified carboxyl groups can bind with heavy metal ions and organic pollutants, making the material effective for water purification. Zhang et al. [256] observed that carboxymethylation disrupted the crystal structure of nanocellulose, converting it from a crystalline form to more amorphous regions. The viscosity of the CMC suspension increased to 88 Pa s, a 363% rise compared to the untreated sample. Electron beam radiation can enhance carboxymethylation. Qin et al. [24] prepared CMC with high carboxyl content through carboxymethylation and mechanical homogenization, demonstrating excellent Cu^2+^ removal performance with a maximum adsorption capacity of 115.3 mg/g. Additionally, carboxymethylated CNF suspension has been shown to be a promising material for electrospun nanocellulose membranes with excellent mechanical and optical properties [183].

### 4.2 Physical modification

#### 4.2.1 Surface adsorption

Surface adsorption involves the use of surfactants to bind molecules to the nanocellulose surface through hydrogen bonding, electrostatic interactions, van der Waals forces, or hydrophobic interactions. Surfactants are commonly categorized into cationic, anionic, and non-ionic types, with examples including SDS, DDAB, and CTAB [257]. Surface adsorption modification typically enhances the properties of nanocellulose. Kai and Jeffrey [222] proposed a method of cationic surfactant adsorption to modify CNC-PLA composite films, improving the dispersibility and interfacial compatibility of nanocellulose within the PLA matrix. This also enhanced the thermal, mechanical, and optical properties of the composites. Nawal et al. [225] compared the rheological behavior of CNFs modified with different surfactants and found that the impact on fluidity and suspension stability depends on the surfactant concentration. Malin and Tizazu [224] modified CNCs with three different cationic surfactants. The modified CNCs exhibited a 100-200% higher contact angle compared to native CNCs, significantly enhancing their hydrophobicity. Furthermore, their dispersibility and corrosion resistance in epoxy resin-based composites were significantly improved.

#### 4.2.2 Ionic liquid welding

Ionic liquid welding is an emerging technology that uses the unique properties of ionic liquids to modify the surface of nanocellulose materials. This method is effective for modifying CNCs and CNFs, with common ionic liquids such as [DBNH][OAc], [BMIm][BF_4_], and [C_16_mim]Br. Ionic liquids impart various beneficial properties to nanocellulose. Shan et al. [228] prepared lubricant additives by integrating ionic liquids into nanocellulose using a hydrothermal method, significantly improving the dispersion stability and lubrication performance. Song et al. [229] modified CNC powder (IL-CNC) with ionic liquids, increasing the thermal degradation initiation temperature of the nanocomposites from 90 °C to 280 °C. The tensile strength and elongation at break also increased with the IL-CNC content. Additionally, ionic liquid modification has been shown to regulate the liquid crystal self-assembly behavior of CNCs [230]. Future research will focus on identifying the most suitable ionic liquids and modification technologies for various applications.

### 4.3 Biochemical modification

#### 4.3.1 Enzymatic reactions

Enzymatic reactions are commonly used for modifying nanocellulose by employing specific enzymes to introduce functional groups. Key enzymes include lipase, laccase, and others. Enzymatic reactions offer milder conditions compared to chemical treatments, and the resulting nanocellulose often shows improved biocompatibility [258]. Hwa Heon Je et al. [232] demonstrated that nanocellulose is a promising carrier for enzyme immobilization, enabling enzyme recovery and reuse via magnetic separation. Zhao et al. [233] produced CNCs via enzyme-catalyzed transesterification, achieving a yield over 78% and enhanced crystallinity and thermal stability. Enzymatic reactions are more efficient and environmentally friendly than traditional acid hydrolysis methods, making them a promising alternative to conventional chemical techniques.

#### 4.3.2 Biological extracts

Biological extracts modification involves covalently bonding bioactive compounds from the extracts to nanocellulose. Common biological extracts include herbal, green tea, and other plant extracts, as well as microbial extracts like bacteria [235]. These extracts are primarily used to modify BNC. While research on the modification of nanocellulose with biological extracts is limited, such modifications typically enhance biocompatibility and antibacterial properties, offering broad application potential in food and biomedicine. Chhavi and Nishi K [235]. modified BNC using extracts from plant leaves and stems. The modified BNC membrane exhibited strong antibacterial activity against *Escherichia coli* and *Pseudomonas aeruginosa*, along with excellent hydrophilicity and porosity, making it suitable for diverse biomedical applications. Furthermore, these products also demonstrate excellent antioxidant properties and have proven effective in food preservation and packaging [236].

## 5 Nanocellulose-based membranes for medical/pharmaceutical applications

The source, type, processing technology, and surface modification strategy of nanocellulose collectively determine the structure, surface properties, and biological performance of the resulting membranes. These factors not only affect membrane morphology, mechanical strength, wettability, and interfacial behavior, but also influence their suitability for specific biomedical and pharmaceutical applications. Nanocellulose can be combined with other nanoparticles or nanosheets to form various nanocomposites. For example, it can be paired with polyvinyl alcohol to produce composite membranes with excellent mechanical properties [38,174,182], or combined with other oxides to create the insulating gel membrane [194]. Additionally, in the field of biological 3D printing, nanocellulose can be combined with sodium alginate as a bio-ink to print 3D scaffold membranes [189]. These nanocellulose-based products, known for their high performance and biocompatibility, have the potential to revolutionize the biomedical and pharmaceutical fields. As research and technology advance, these products will further drive progress in biomedicine. Fig. 10 illustrates the applications of nanocellulose products in biomedicine.

### 5.1 Tissue engineering scaffolds

Tissue engineering scaffolds are widely used for repairing, regenerating, and replacing damaged tissues and organs, including skin, blood vessels, nerves, skeletal muscle, and the heart [259]. Tissue engineering scaffolds generally exhibit good biocompatibility, high porosity, and adequate mechanical strength. These properties can be further enhanced by surface modification or polymer composite techniques to increase cell activity [[260], [261], [262], [263]]. Scaffold membranes for tissue engineering can be fabricated using various methods, including solution casting, electrospinning, 3D printing, and combined molding. Scaffolds produced by different techniques exhibit varying porosity, surface area, and mechanical strength [264]. Nanocellulose-based tissue engineering scaffolds can be fabricated into composite materials, such as films, hydrogels, and aerogels, to fulfill specific medical requirements. Studies have shown that nanocellulose scaffolds can replicate the mechanical and structural properties of natural tissues, improving their effectiveness in regenerative medicine [265]. Amaral et al. [266] produced a Poly(globalide)/regenerated cellulose bilayer membrane using a simple layer-by-layer casting process. Human keratinocytes exhibited good metabolic activity on this membrane, which can be used for epidermal regeneration culture. Nanocellulose scaffolds with tailored porosity and mechanical properties have been shown to enhance cell adhesion and tissue integration. Rekha Unni et al. [267] prepared nanocellulose scaffold membranes with a tensile strength of up to 69 MPa using a casting method. The 1% PEG-plasticised film maintained a constant percentage of cell viability during 5 days of cell culture, with a haemolysis rate of less than 1%, demonstrating good cytocompatibility and hemocompatibility. Chemical modification can further improve the interaction between cells and materials, promote cell adhesion, and facilitate the delivery of growth factors or other bioactive molecules [259].

### 5.2 Biosensor

The exceptional properties and versatility of nanocellulose membranes make them ideal materials for biosensing. Nanocellulose-based biosensors exhibit excellent optical transparency, thermal stability, flexibility, and mechanical strength. Mahnaz M et al. [268] developed a sensitive electrochemical cholesterol biosensor by immobilizing cholesterol oxidase on ionic liquid-modified CNC. This biosensor can monitor cholesterol concentrations in the range of 0.001–12 mM with minimal interference. The resulting sensors effectively detect laccase and demonstrate high signal stability. Biosensors typically require a large amount of electrolyte solution to generate analytical signals, and nanocellulose membranes can serve as ideal sensor substrates. Joyati et al. [269] used nanocellulose as an immobilized substrate for xanthine oxidase to fabricate an electrochemical biosensor for detecting xanthine in fish. The sensor had a detection linear range of 3–50 μM, a detection limit of 47.96 nM, and a sensitivity of up to 5281 μA mM^−1^ cm^−2^. Furthermore, chemically modified nanocellulose composites demonstrate exceptional sensitivity and specificity. These products can be embedded in sensing systems on human skin for real-time analysis [257]. Xu et al. [270] used TEMPO-oxidized nanocellulose and sulfonated multi-walled carbon nanotubes to prepare flexible electronic skin, which exhibited high sensitivity of 4.4 kPa^−1^ and mechanical strength of up to 184 MPa. They are expected to replace traditional sensors based on plastic, glass, or paper platforms in the future [271].

### 5.3 Drug delivery systems

Nanocellulose composite materials have recently gained significant attention in drug delivery systems due to their high capacity for loading and binding active pharmaceutical ingredients, which allows for effective drug release control [272]. Factors influencing drug delivery systems include drug carriers, loading rates, and release rates, with the specific surface area of the carrier being crucial in controlling the drug release rate [272]. Nanocellulose-based drug delivery systems can generate specific release profiles and enhance the bioavailability of therapeutic drugs. Anirudhan et al. [273] used folic acid-coupled nanocellulose to develop a strategy for controlled pH-triggered curcumin delivery, achieving a drug loading capacity of 89.2%, making it applicable for cancer treatment. Nanocellulose films loaded with antibiotics have shown promising results in wound care applications, offering localized and sustained drug release [274]. Chemically modified nanocellulose can enhance barrier properties and control drug release kinetics in drug delivery systems. Garrido-Miranda et al. [275] fabricated a composite membrane consisting of nanocellulose and nanoporous silica particles. Not only were the mechanical properties of the membrane significantly enhanced (elastic modulus increased by 70% and elongation increased by 372%), but the methylene blue dye released by the membrane effectively inhibited the growth of *Escherichia coli* and *Staphylococcus aureus*. The incorporation of antimicrobial agents or other therapeutic compounds enables targeted drug delivery.

### 5.4 3D printing

In recent years, 3D printing, also known as additive manufacturing, has relied on 3D computer-aided design to create complex new materials. Nanocellulose can form hydrogels with high zero-shear viscosity, and its excellent mechanical and rheological properties make it an ideal material for 3D printing [276]. Recent research has shown that CNC-based nanocomposite materials can enhance the mechanical and thermal properties of 3D printed resin materials [277]. Additionally, nanocellulose can be used as a raw material for 3D bio-inks, cross-linking with alginate to print grids with excellent mechanical stability and biocompatibility. These grids have potential applications in wound dressings and cardiovascular tissue engineering [178,179]. Florian Lackner et al. [278] used 4-axis 3D printing to manufacture nanofiber cellulose tubular scaffolds that mimic pig aortas. The fiber orientation can be selectively distributed according to different printing modes, and the scaffolds maintain high compressive strength, capable of withstanding water pressure of up to 500 mmHg^−1^. Despite the high calcium ion concentration limiting the material's haemostatic properties, the scaffold maintained over 90% cell viability for both HEK293 and HUVEC cells. Furthermore, 3D composite materials based on nanocellulose can be used to create various medical implants, such as cochlear implants, tracheal tubes, cardiovascular devices, and prosthetics [279]. Compared to metals and ceramics, nanocomposite-printed implants offer superior shape fidelity and biodegradability. Researchers are actively developing optimal nanocellulose bio-inks and optimizing 3D printing parameters to create high-performance nanocellulose 3D printing products for commercial applications.

### 5.5 Antibacterial applications and clinical translation limitations

#### 5.5.1 Hierarchical antibacterial mechanisms

Nanocellulose-based membranes are receiving increasing attention for infection control in applications such as tissue scaffolds and wound healing. Their antibacterial performance can be considered from three aspects: intrinsic antibacterial activity, incorporation of antimicrobial agents, and physical barrier effects. First, nanocellulose exhibits only limited intrinsic antibacterial activity. Pure cellulose generally does not exert strong direct bactericidal effects against common wound pathogens. Therefore, nanocellulose generally requires functionalization or the incorporation of bioactive agents to effectively combat wound infections [278].

Second, the most effective antibacterial performance of nanocellulose membranes typically arises from the incorporation or loading of antimicrobial agents. These may include antibiotics, bioactive molecules, cationic polymers, graphene-based additives, metal ions, or other antimicrobial compounds. The abundance of hydroxyl groups on the cellulose surface provides versatile binding sites for the immobilization of these agents [280]。 Recent advances have been reported in the design of nanocellulose-derived scaffold membranes with improved antibacterial performance and infection-control properties. For example, Rekha et al. [279] incorporated 5% curcumin into a PEG-plasticised nanocellulose matrix, where multiple hydrogen-bonding interactions between curcumin and cellulose monolayers facilitated the development of a multifunctional biodegradable scaffold, achieving a minimum inhibitory concentration against *Staphylococcus aureus* of 885.76 μg/mL. Similarly, Maryam et al. [281] fabricated electrospun nanofiber membranes by reinforcing PLA/gelatin solutions with modified CNCs, resulting in an average bacterial growth inhibition rate of 68.3% ± 36.5%. This strategy not only enhanced antibacterial efficacy but also improved the mechanical strength and hydrophobicity of the membranes.

In addition, the physical barrier effect plays a crucial role in wound protection. From a structural perspective, the nanofibrous architecture of nanocellulose scaffolds confers a high specific surface area, large porosity, and excellent hydration capacity [278]. These features provide abundant sites for the release of loaded antibacterial agents, while the hydrated state promotes intimate interaction with body fluids and facilitates the diffusion of bioactive molecules. At the same time, the interconnected fibrous network acts as a physical barrier to restrict bacterial migration [282]. Previous studies have reported that porous nanocellulose membranes can effectively prevent bacterial penetration while maintaining a moist and oxygen-permeable environment conducive to tissue repair [83,280,283].

#### 5.5.2 Challenges in clinical translation and large-scale production

Despite the encouraging progress made in the aforementioned research, the cost-effectiveness and scalability of production remain major barriers to the industrial application of nanocellulose scaffold membranes. First, most current research is largely confined to in vitro assays and animal models, with no clear regulatory guidelines for their clinical use. Second, potential cytotoxicity and long-term stability are critical parameters that must be rigorously validated before clinical translation [270,271].

Furthermore, large-scale production of nanocellulose-based biomedical products must address challenges related to cost, cytocompatibility, process stability, and regulatory standardization. Finally, robust quality-control systems for large-scale manufacturing will be indispensable to support their eventual transition into clinical practice. This requires validated sterilization protocols, long-term storage stability, and well-defined quality control standards addressing residual reagents, endotoxin risk, and structural uniformity. Accordingly, future development should focus not only on enhancing biological performance, but also on establishing scalable, cost-effective, and standardized manufacturing strategies that meet clinical regulatory requirements and enable industrial translation.

## 6 Design and optimization of novel nanocellulose composite membranes

Nanocellulose membranes, with their diverse properties, are applicable in various fields, including packaging, biomedicine, electronic devices, filtration, dynamic information storage, and food additives. Nanocellulose membranes must meet strict requirements to ensure their effectiveness, safety, and compatibility with biological systems in various applications. Most importantly, the design of nanocellulose membranes should follow a comprehensive approach, treating raw material sources, processing technology, functionalization strategies, final membrane structure, and target applications as an interconnected system.

**Fig. 12:**
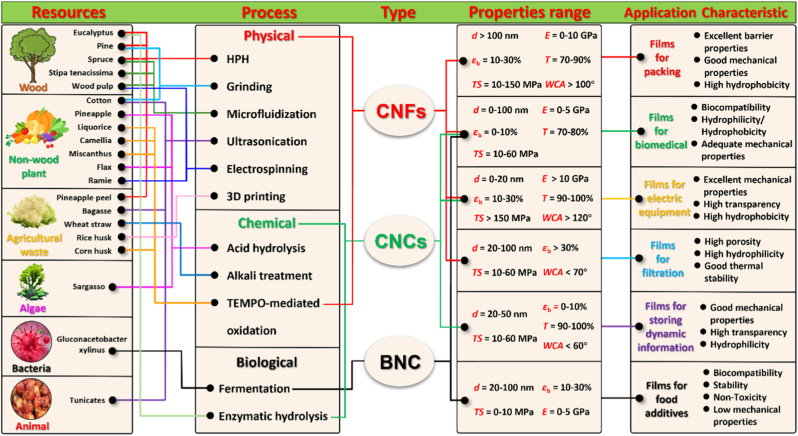
Candidate materials and preparation of nanocellulose membranes for different applications (Symbols: *d* = Fiber diameter, *TS* = Tensile strength, *ε*_b_ = Elongation at break, *E* = Young's modulus, *T* = Optical transmittance, *WCA* = Water contact angle).

Key parameters, including diameter, porosity, tensile strength, elongation at break, Young's modulus, optical transparency, biocompatibility and water contact angle, are crucial for film performance. A combination of these parameters can be customized for specific functionalities to create membranes suitable for various applications. Additionally, the source of nanocellulose and the preparation method influence the product's characteristics. Fig. 12 illustrates the design process for nanocellulose membranes in various applications. From the figure, it can be observed that wood-based materials and tunicates primarily generate CNFs through physical processing, whereas certain non-wood-based plants (including agricultural waste) can yield CNCs via chemical treatments and enzymatic hydrolysis. Membrane products derived from CNFs exhibit a broad range of properties, characterized by good toughness and suitable pore size distribution, making them widely applicable in packaging, filtration, and biomedical scaffolds. In contrast, CNC-based membranes demonstrate higher stiffness and superior optical properties compared to CNF-based ones, enabling their use in advanced electronic equipment and dynamic information storage [284]. Meanwhile, BNC membranes produced through bacterial fermentation, although lacking high mechanical strength, possess exceptional purity and biocompatibility, which makes them suitable for applications as food additives and biomedical materials.

Nanocellulose membranes used in biomedical applications possess suitable physical properties (*d* = 0-100 nm, *TS* = 10-60 MPa, *ε*_b_ = 0-10%, *E* = 0-5 GPa, *T* = 70-80%) to ensure efficacy, safety, and compatibility with biological systems. Recently, more researchers have focused on developing high-performance nanocellulose membranes through various modification methods and composite materials [30,59,157,170,198,285]. The design of biomedical nanocellulose membranes should shift from descriptive material selection strategies to a structure-oriented optimization framework. Within this framework, the material source determines the available nanoscale structural units, the fabrication process defines the membrane structure, functionalization controls the interface, and the final membrane structure determines its biological properties. For biomedical scaffold membranes, the most relevant structural objectives typically include: (i) suitable pore size and interconnectivity to facilitate cell infiltration and nutrient exchange; (ii) sufficient wet mechanical strength and flexibility to facilitate manipulation and tissue adaptation; (iii) controllable wettability and surface chemistry to facilitate protein adsorption and cell adhesion; (iv) stable but tunable degradation or swelling behavior, and, where necessary, integrated antibacterial or drug delivery functions.

This paper presents an innovative design-oriented platform based on nanoscale regenerated cellulose membranes prepared by electrospinning and pre-evaluates its applicability. Through this platform, regenerated cellulose, nano-reinforcing materials, and functional additives can be systematically combined to regulate membrane structure and enhance biomedical performance.

### 6.1 Nanocellulose composite membranes based on regenerated cellulose

The performance of polymer films depends on their mechanical properties, including tensile strength, Young's modulus, and elongation at break. A membrane is a material that permits solvents and specific solutes to pass through while blocking others, thus enabling mass transfer. It is primarily used in dialysis, ultrafiltration, and separation, where porosity, permeability, and retention rate are key performance indicators. Compared to commonly used commercial biomedical membranes, such as expanded polytetrafluoroethylene (ePTFE) [286] and polyurethane (PU) [287], regenerated cellulose membranes offer excellent biodegradability, hydrophilicity and a high density of functional groups. These functionalities enable bioactive modification without the need for harsh plasma treatments, making them an environmentally friendly alternative to petroleum-based polymers [288]. Their abundant hydroxyl groups facilitate modification, allowing functional additives (such as antimicrobial agents and conductive materials) to be introduced during the dissolution stage, achieving uniform molecular-level composites [261,262,289].

The uniqueness of regenerated cellulose composite membranes lies in the fundamental structural reorganization that occurs during the dissolution and regeneration process. This transformation, typically at the micron scale, converts cellulose I to cellulose II through a “bottom-up” approach. The rearrangement of the internal hydrogen-bonding network significantly reduces crystalline rigidity and enhances molecular chain flexibility [103]. Compared to conventional natural cellulose membranes, regenerated cellulose eliminates interfacial defects during the dissolution and regeneration process, exhibiting higher polymerization degree, flexibility, and chemical stability. It also addresses the issue of difficult dissolution in natural cellulose, improving solution uniformity and flowability, thereby enhancing the stability of the membrane-forming process [290]. Membranes with pore sizes smaller than 10 μm can also serve as dialysis membranes, removing low molecular weight substances via solute diffusion [291].

At this stage, regenerated cellulose can be converted into ECNF using electrospinning technology [292,293]. Electrospinning technology preserves cellulose's optimal characteristics while enhancing its surface area-to-volume ratio, mechanical properties, and enabling surface customization of bulk materials [294]. However, poor solubility remains a significant challenge in the electrospinning of native nanocellulose. CNF and CNC typically require blending with polymeric additives or carriers such as polyvinyl alcohol (PVA) or polylactic acid (PLA) to form a spinnable solution, which often suffers from limited dispersibility, high viscosity, and phase incompatibility. In contrast, regenerated cellulose is readily soluble in a variety of green solvent systems, enabling the formation of stable, homogeneous spinning solutions without the need for additional carriers. This substantially improves electrospinning processability and fiber uniformity. Furthermore, regenerated cellulose exhibits greater molecular chain flexibility due to its lower crystallinity and altered hydrogen bonding structure, which facilitates the formation of continuous, bead-free, and highly porous nanofibrous networks. These structural features confer enhanced mechanical flexibility to the resulting membranes, making electrospun regenerated cellulose particularly attractive for applications in soft tissue engineering, wound healing, and localized drug delivery.

In the biomedical field, flexible electronic devices based on ECNFs are prepared using new dissolution techniques and derivatives of natural cellulose. This results in epidermal electrodes, patches, and films with orderly nanoscale mesh and porous structures [274,275,277]. These products exhibit excellent biocompatibility, breathability, self-adhesiveness, and low impedance, with promising applications in biomedical fields, photothermal responsiveness, and energy signal storage. To expand the applications of cellulose nanomaterials, future research should focus on cellulose dissolution and regeneration, chemical or physical modifications, and structural design, as these factors are crucial to their performance and functionality [295].

### 6.2 Preparation process of regenerated nanocellulose membranes

#### 6.2.1 Dissolution and regeneration of cellulose

Cellulose dissolution is a critical step in the preparation of regenerated cellulose. The traditional process uses sodium hydroxide and carbon disulfide to form cellulose xanthate esters, which are then dissolved in NaOH solution, often resulting in environmental issues [296]. In recent decades, new, simple, low-toxicity, and environmentally friendly cellulose solvents have been developed, including N-methylmorpholine-N-oxide (NMMO), LiCl/N,N-dimethylacetamide (LiCl/DMAc), ionic liquids, and alkali/urea solutions [[297], [298], [299], [300]].

The direct dissolution of cellulose in the more efficient and environmentally friendly NMMO solvent is known as the lyocell process, commonly used in the production of lyocell fibers. The N-O bond in the NMMO molecule is highly polar, which enhances its solubility in cellulose. Additionally, NMMO can be recovered and recycled, minimizing waste and making the process more environmentally friendly. Mao et al. [298] solubilized cellulose using the NMMO system to prepare regenerated cellulose membranes with a homogeneous structure, high crystallinity, and good mechanical properties through coalescence and regeneration, which can be used for the dehydration of isopropanone.

Compared to other solvent systems, LiCl/DMAc is a well-established, non-derivatized, non-aqueous solvent that can solubilize large molecular weight cellulose without causing degradation in solution [301]. The combined effect of the basicity of the Cl^−^ ion and the spatial resistance of the [Li(DMAc)_4_]^+^ unit breaks the hydrogen bond between two cellulose chains at the non-reducing end of the cellulose molecule [302]. Activation pretreatment enhances cellulose accessibility to the solvent, accelerating the dissolution rate [303]. Studies show that solvent-treated microcrystalline cellulose with LiCl/DMAc can be solubilized up to 16 wt%, and its nanoporosity can be effectively improved [99,304]. Zhu et al. [305] oriented the aggregation structure of solubilized cellulose in the LiCl/DMAc system, producing a high-performance film with tensile strength of 145.9 MPa and light transmittance of 86.2%, enhancing the versatility of such products. However, small solvent molecules in LiCl/DMAc often remain in the regenerated cellulose film, making them difficult to remove and leading to degradation of the film's performance. Therefore, optimized methods have been developed to maximize the removal of residual DMAc and Li^+^ [306].

Ionic liquids, a new class of solvents with unique properties, have garnered attention for their potential in dissolving cellulose. The anions in ionic liquids disrupt the hydrogen bonding network between cellulose molecules, and the dissolution process can be optimized by adjusting the synergistic effects of anions and cations [307]. Compared to NMMO and LiCl/DMAc, ionic liquids, especially [Emim]Ac, are the most effective solvents for dissolving cellulose, particularly bleached softwood sulfite dissolving pulp [308]. Chen et al. [309] used the ionic liquid [BMIM]Cl to prepare regenerated wheat straw cellulose membranes with tensile strength of 170 MPa and elongation at break of 6.4%, demonstrating good permeability and potential for separation membrane applications.

The solvent dissolution of cellulose in alkali/urea aqueous solution is an environmentally friendly, low-temperature method, first demonstrated by Zhang et al. [310] in 2004, by adjusting the NaOH and urea components and controlling the solvent temperature (pre-cooled to below −10 °C) for 5 min at room temperature. The presence of alkali hydrate and urea hydrate in the aqueous solution is essential for cellulose dissolution at low temperatures, with LiOH demonstrating greater dissolving ability than NaOH [311]. A variety of new regenerated cellulose products for different applications have been successfully prepared using this system, including membranes, microspheres, hydrogels, and biodegradable transparent, fluorescent, and long-afterglow photoluminescent films and functional materials [312]. However, the low solubility of cellulose processed through the alkali/urea aqueous solution system, the poor stability of the spinning solution, and the need to improve the strength of finished fibers leave room for future research.

#### 6.2.2 Membrane-forming methods

Most membrane materials are processed using the phase separation method. This method, introduced in the 1960s, has become the most commonly used preparation technique due to its ease of operation and broad range of applications [295,313]. It involves casting a film solution that exchanges solvents and non-solvents with the surrounding environment, causing a phase change in the original solution, which solidifies into a film in a different phase. The phase separation method includes four main techniques: thermally induced phase separation [314], immersion precipitation [315], solvent evaporation [316], and gas-phase precipitation [317]. The immersion precipitation method is primarily used for preparing natural polymer membranes, such as regenerated cellulose membranes via the viscosity method.

The preparation of regenerated cellulose membranes is influenced by factors such as the composition of the coagulation bath, coagulation temperature, additives, and post-treatment conditions, which significantly affect the structure and properties of the materials [104]. Zhang et al. [318] investigated the effect of different coagulation conditions on the properties of regenerated cellulose membranes. They found that membranes prepared using an acid coagulation bath had smaller pore diameters and narrower pore size distributions, while those prepared with organic solvents showed the opposite trend. Additionally, the mechanical properties of cellulose membranes prepared with acid and salt coagulation baths differed. Regenerated cellulose membranes prepared with H_2_SO_4_/Na_2_SO_4_ exhibited the highest transmittance and mechanical properties. H_2_SO_4_/Na_2_SO_4_ is also the most common curing bath for preparing regenerated cellulose membranes in alkali/urea systems [301,302].

Temperature significantly influences both the regeneration process and the properties of recycled cellulose membranes [319]. As the curing bath temperature increased from 10 °C to 60 °C, the average pore size and water permeability of the recycled membranes also increased. IR and UV results showed that intermolecular forces in the recycled cellulose membranes were stronger at lower temperatures. In addition to NaOH/urea, regenerated cellulose from LiOH/urea and NaOH/thiourea exhibited a similar pattern: membranes prepared at lower curing bath temperatures had a more regular and denser structure, resulting in higher mechanical strength and light transmittance [320].

During polymer solubilization, additives can alter the chain conformation and dispersion state of polymers, thereby affecting the structure and properties of polymeric membrane materials. Polymer additives can increase the solubility of insoluble drugs in exclusion membranes, though they may also reduce certain permeability [321]. Liu et al. [322] found that adding N-methylimidazole to ionic liquid-solubilized regenerated cellulose membranes effectively reduced the DP loss of regenerated cellulose. This was attributed to the neutralization of the alkalinity of N-methylimidazole with the acidity of ionic liquids, inhibiting cellulose depolymerization. ZnO can increase the maximum solubility of regenerated cellulose in the NaOH/urea system. The regenerated cellulose membranes prepared with NaOH/urea/ZnO and H_2_SO_4_ coalescence baths exhibit higher light transmittance, thermal stability, and tensile strength [323].

Post-treatment conditions also affect regenerated cellulose membranes. Common methods include orientation, drying, and distillation. Orientation is a commonly used technique to improve material properties by forming an ordered supramolecular structure and enhancing intermolecular forces. Tensile orientation typically results in ordered nanofibrous structures in the film or gel, enhancing the tensile strength and toughness of cellulose [103]. Ye et al. [324] proposed a structural densification method using preorientation-assisted dual cross-linking to prepare strong, ultra-transparent regenerated cellulose films with anisotropic nanofibrous structures from dissolved cellulose in alkaline/urea solutions. These films are suitable for next-generation packaging, flexible electronics, and optoelectronic devices. Drying removes solvents from the solution and forms stable film structures. Different drying methods affect regenerated cellulose films in various ways. Over-drying may cause cracking and shrinkage of regenerated cellulose membranes, while infrared drying has less impact on membrane properties compared to oven drying [325]. Zeng et al. [326] treated wet-spun fibers with freeze-drying and supercritical drying methods. They found that freeze-dried fibers had a porous surface, while supercritical-dried fibers had a smooth surface and internal nanopores. Both methods enhanced the porosity of regenerated cellulose, promoting further modification and functional optimization. Distillation is an effective separation technique. A complex distillation process using an isopropylacetone/water mixture can improve membrane quality by minimizing residual impurities and ion concentrations in regenerated cellulose membranes [306].

### 6.3 Nanostructured regenerated cellulose composite membranes

The combination of regenerated cellulose with inorganic and organic substances can be blended and modified to create nanoscale composite membranes. This development enhances the membranes' optical, electrical, magnetic, and bioactive properties, marking an important advancement in the field of natural polymer materials [103]. These composite membranes have gained significant attention due to their versatility, sustainability, and potential for advanced functionality. Various nanofillers and reinforcements, such as CNWs, CNFs, nanoparticles (e.g., SiO_2_ and TiO_2_), graphene oxides (GOs), polylactic acid (PLA), and other polymers, have been used to prepare regenerated cellulose-based composites [[327], [328], [329], [330], [331]]. Advanced techniques such as electrostatic spinning, phase transition, and layer-by-layer assembly enable precise incorporation of nanoscale fillers, optimizing membrane structures.

**Fig. 13:**
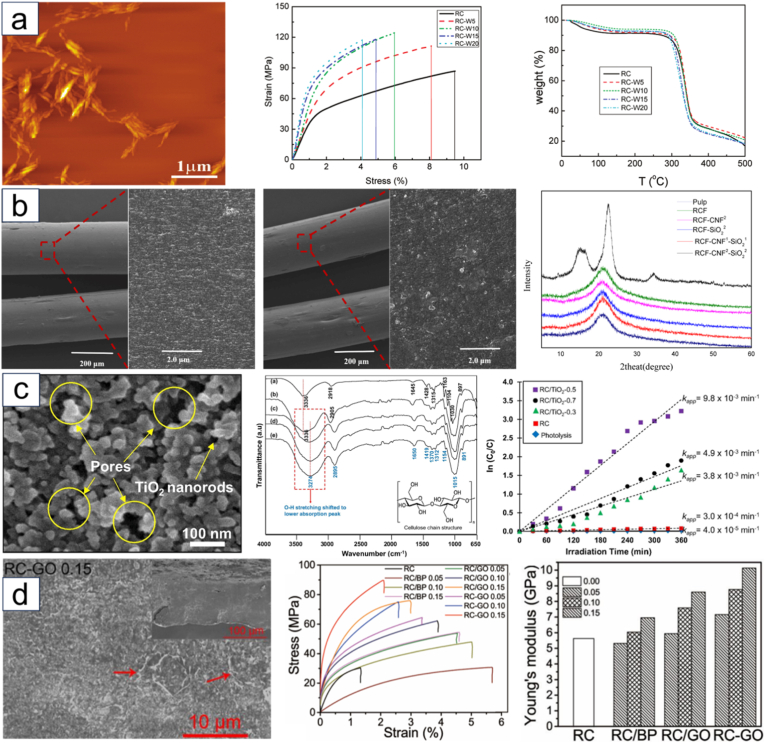
(a) AFM topography image of cellulose whiskers after drying on a mica surface; Stress-strain curves of all-cellulose composite films and pure regenerated cellulose films; Temperature dependence on weight loss (TGA curve) of RC films and composite films. (Adapt from Ref. [327]). (b) SEM images of the regenerated cellulose fibers: RCF-CNF_2_-SiO_2_^2^ at × 1000; XRD analysis of the regenerated fibers with or without nanomaterials versus control cellulose pulp (Adapt from Ref. [328]). (c) FESEM images of RC/TiO_2_-0.5; FTIR spectra; Kinetic of disappearance of phenol by RC/TiO_2_ nanocomposite membrane with different TiO_2_ loading (0–0.7 wt%) under UV irradiation (Adapt from Ref. [329]). (d) SEM image of the top surfaces and the cross-sections of the RC nanocomposites incorporated with GO and BP nanosheets; Mechanical properties of RC film and the composites as a function of different additive loadings of BP and GO (Adapt from Ref. [330]).

Qi et al. [327] prepared composite films by adding varying amounts of CNWs to a regenerated cellulose solution using temperature-controlled solubility. As shown in Fig. 13(a), CNWs with an average diameter of 21 nm were dispersed on the film surface in the form of needles. A 5-10 wt% CNW content significantly improved the mechanical and thermal properties of the composite film, with the tensile strength reaching 124 MPa. Xue et al. [328] found that adding CNFs and SiO_2_ particles effectively filled the pores of regenerated fibers dissolved in the ionic liquid system. The simultaneous addition of CNFs and nano-SiO_2_ resulted in a smooth and flat material (Fig. 13(b)). XRD results showed that CNFs and SiO_2_ did not affect the crystal structure of cellulose during regeneration. The tensile strength of the composite film with 1% CNF and 1% SiO_2_ increased by 47.46%, reaching 229.78 MPa, despite a decrease in crystallinity. Liu et al. [332] proposed using a LiOH/urea solvent system combined with TEMPO-oxidized CNFs and GOs as fillers to prepare high-performance regenerated cellulose membranes. The incorporation of 5 wt% CNF or 0.4 wt% GO significantly improved the mechanical properties, primarily by filling defects and enhancing stress transfer efficiency. Mohamad et al. [329] used the phase conversion method to prepare photocatalytically active regenerated cellulose membranes doped with TiO_2_ nanorods, as shown in Fig. 13(c). The composite membranes exhibited uniformly dispersed TiO_2_ nanorods and a porous structure. The incorporation of TiO_2_ nanorods enhanced hydroxyl group absorption peaks, indicating strong hydrogen bonding interactions. The optimal photocatalytic performance was achieved with 0.5 wt% TiO_2_ nanorods, showing a 96.6% phenol degradation rate under UV irradiation. Zhang et al. [330] investigated the effect of GO and black scale (BP) as reinforcing agents in regenerated cellulose composite films, as shown in Fig. 13(d). GO and BP nanosheets made the composite film's fracture surface flatter, improving the interfacial bonding strength. The experiments showed that GO and BP at 0.15 wt% exhibited the best mechanical properties, with tensile strength and Young's modulus of 89.9 MPa and 10.13 GPa, respectively. Xu et al. [331] used the ionic liquid 1-butyl-3-methylimidazole acetate [bmim]Ac/N,N-dimethylformamide (DMF) system to overcome cellulose and polylactic acid (PLA) incompatibility. The tensile strength of the cellulose/PLA composite film was 52% higher than that of pure cellulose, and the product exhibited good biocompatibility, biodegradability, and cell viability, showing strong potential for biomedical applications.

## 7 Challenges and prospects

Despite the rapid advancements of nanocellulose-based scaffold membranes in the biomedical field, several critical issues continue to impede their clinical translation [6,84]. In practice, the performance requirements for biomedical scaffold membranes are often more stringent than those of conventional commercial membranes. A central challenge in this field lies in the necessity for scaffolds to simultaneously satisfy multiple, often mutually incompatible requirements, including porosity, mechanical strength, cytocompatibility, biodegradability, sterility, and scalable productivity. In fact, these requirements are difficult to optimize concurrently. For example, membranes with higher porosity can enhance permeability, cell infiltration, and nutrient transport, but often at the expense of mechanical strength. Similarly, enhanced biofunctionalization may improve interfacial compatibility, yet the potential increase in cytotoxicity introduces additional regulatory concerns for clinical application.

From the perspective of clinical translation, the field currently lacks unified performance metrics, benchmark datasets and regulatory-oriented evaluation protocols. For scaffold membranes intended for clinical use, future assessment frameworks must extend beyond basic morphology and dynamic mechanical properties. They should encompass wet-state stability, sterilization tolerance, protein adsorption, degradation profiles, and long-term biosafety. From the standpoint of cost and scalability, significant challenges remain for industrial implementation. Expenses associated with high-purity raw materials, fiber extraction, solvent recovery, and post-modification techniques collectively inflate production costs. Plant-derived systems are generally more amenable to scale-up, yet issues related to batch consistency and purification persist. In contrast, bacterial nanocellulose offers higher purity, but its fermentation and downstream processing remain costly. Consequently, many high-performance membranes are still difficult to translate into economically viable biomedical products.

Overall, future progress in this field will depend on a shift from isolated material optimization toward integrated design strategies [333]. In this context, rege'nerated nanocellulose composite membranes are emerging as a promising design framework for next-generation biomedical scaffold membranes. Their significance lies not merely in replacing conventional nanocellulose membranes, but in expanding the design space of cellulose-based biomedical materials. Conventional nanocellulose membranes are widely favored due to their inherent fibrillar structure, high crystallinity, and robust mechanical strength. However, when uniformity, structural tunability, multi-component compatibility, and customizable fabrication become critical design priorities, regenerated systems may offer distinct advantages. If scalable processing routes can be developed to convert regenerated cellulose into nanoscale structures, thereby compensating for the loss of strength associated with structural reorganization, regenerated nanocellulose composite membranes could evolve into versatile platforms for tailored wound dressings, soft tissue scaffolds, drug delivery systems, and other advanced biomedical interfaces.

## 8 Conclusions

In conclusion, nanocellulose scaffold membranes provide a versatile and sustainable platform for biomedical applications. Their inherent biocompatibility, tunable mechanical properties, and multifunctionality represent a highly promising next-generation biomedical material. However, in order to successfully transition nanocellulose scaffold membranes from laboratory research to large-scale product applications, it is necessary to further explore how their source, pretreatment strategies, processing techniques, and chemical modifications directly affect the key performance characteristics of the product.

Among the sources of nanocellulose discussed in this paper, bacterial nanocellulose standed out for its high purity and excellent water-holding capacity, making it particularly suitable for applications in soft tissue scaffolds and wound dressings. In contrast, plant-derived CNFs and CNCs exhibited higher mechanical strength and scalability, but typically required more stringent pretreatment strategies to remove residual impurities and ensure material biocompatibility. Different pretreatment strategies could significantly influence the morphology, crystallinity, and dispersibility of nanocellulose. Therefore, the selection of pretreatment methods must balance the mechanical properties, permeability and surface functionality required for cellular interaction in the intended application.

Compared to many existing reviews, the unique contribution of this review lies in its integrative and application-oriented perspective. This review explored the balanced combination of factors influencing the properties of nanocellulose scaffold membranes, from raw material sources and processing to chemical modification. The synergistic impact of source selection, fabrication techniques, and functionalization strategies must be explored to achieve a balanced design for nanocellulose-based scaffold membranes. Furthermore, this review identified a central challenge in the field: biomedical scaffold membranes must reconcile inherently competing requirements, including porosity, mechanical stability, cytocompatibility, hemocompatibility, and cost-effectiveness.

In this context, the development of regenerated nanocellulose composite membranes emerged as a promising future direction for customized and tunable design. Future research must integrate material selection, scalable processing, tailored functionalization, standardization, and clinical requirements into a coherent design framework. Only through such integration can nanocellulose-based scaffold membranes evolve from laboratory-scale materials into clinically relevant and industrially viable biomedical products.

## CRediT authorship contribution statement

**Hanyuan Chen:** Data curation, Formal analysis, Investigation, Writing – original draft. **Yanqun Huang:** Data curation, Formal analysis. **Feng Wang:** Data curation, Investigation. **Ziyan Fu:** Data curation, Investigation. **Ao Li:** Data curation, Investigation. **Malaz Abdelsadig:** Data curation, Investigation. **Mark Brassil:** Conceptualization, Data curation. **Yan Xia:** Conceptualization, Data curation, Formal analysis. **Bei Zhou:** Data curation, Investigation. **Guanben Du:** Conceptualization, Investigation, Supervision. **Mizi Fan:** Conceptualization, Funding acquisition, Supervision, Writing – review & editing.

## Declaration of competing interest

The authors declare that they have no known competing financial interests or personal relationships that could have appeared to influence the work reported in this paper.

## Data Availability

Data will be made available on request.
